# Challenges and benefits of using unstructured citizen science data to estimate seasonal timing of bird migration across large scales

**DOI:** 10.1371/journal.pone.0246572

**Published:** 2021-02-04

**Authors:** Nadja Weisshaupt, Aleksi Lehikoinen, Terhi Mäkinen, Jarmo Koistinen

**Affiliations:** 1 Finnish Meteorological Institute, Helsinki, Finland; 2 Finnish Museum of Natural History, University of Helsinki, Helsinki, Finland; Universite du Quebec a Chicoutimi, CANADA

## Abstract

Millions of bird observations have been entered on online portals in the past 20 years either as checklists or arbitrary individual entries. While several hundred publications have been written on a variety of topics based on bird checklists worldwide, unstructured non-checklist observations have received little attention and praise by academia. In the present study we tested the suitability of non-checklist data to estimate key figures of large-scale migration phenology in four zones covering the whole of Finland. For that purpose, we analysed 10 years of ornithological non-checklist data including over 400 million. individuals of 115 bird species. We discuss bird- and human-induced effects to be considered in handling non-checklist data in this context and describe applied methodologies to address these effects. We calculated 5%, 50% and 95% percentile dates of spring and autumn migration period for all species in all four zones. For validation purposes we compared the temporal distributions of 43 bird species with migration phenology from standardized long-term ringing data in autumn of which 24 species (56%) showed similar medians. In a model approach, non-checklist data successfully revealed latitudinal migration progression in spring and autumn. Overall, non-checklist data proved to be well suited to determine descriptors of migration phenology in Northern Europe which are challenging to attain by any other currently available means. The effort-to-yield ratio of data processing was commensurate to the outcomes. The unprecedented spatiotemporal coverage makes non-checklist data a valuable complement to current migration databases from bird observatories. The basic concept of the present methodology is applicable to data from other bird portals, if combined with local field ornithological knowledge and literature. Species-specific descriptors of migration phenology can be potentially used in climate change studies and to support echo interpretation in radar ornithology.

## Introduction

Technological advances in the past 20 years have enabled the creation of online bird portals dedicated to the collection of casual daily visual and acoustic field observations. Prominent examples of such online databases are the *Ornitho*, *Observation* and *BirdTrack* platforms in Europe (for an overview see [1]) or the US-American *eBird*. The general idea of these portals is to report sightings of birds, with species, number of birds and location as minimum information required and optionally additional details, such as the birds’ age, sex, behaviour, observation time and so on. Through the widespread network of observers, millions of observational reports accumulate across vast areas throughout the year, providing an unprecedented trove of information. This wealth of observations could be potentially used for scientific research about avifaunal dynamics, as a complement to traditional monitoring programs through bird surveys and ringing by volunteers and scientists [2]. Despite more than 300 publications based on *eBird* data (https://ebird.org/about/publications/), the use of such bird repositories other than *eBird*, is still hesitant in the scientific community.

Hesitation to use these data portals is often linked to quality concerns and data availability [3, 4], but probably also with unfamiliarity with and unawareness of the data portals. Quality concerns relate mainly to variable observer expertise and heterogeneity in data collection, so-called observer bias, which can lead to false positive or negative records [2, 5]. Observer bias is a well-known issue in observational field data, also those collected by professionals, and can originate from the observers’ level of ornithological knowledge and experience, species attractiveness (e.g. rare vs. abundant species, first appearance in spring), hearing and visual capacity, motivation and dedication depending on environmental conditions and so on [6–8].

Theoretically, many of these observer biases can be controlled prior to data analysis by a careful study design, preparation, and training of the samplers before the field work starts [9]. Various publications and manuals address potential and actual pitfalls of using citizen science data in general, also outside ornithology, and instruct on how to obtain homogeneous data and reliable results [10, 11]. However, in case of bird portals, where data is gathered continuously and unawarely of some potential research interest, the researchers face the fait accompli of the pool of both observations and data collectors with an unknown amount of bird-, human- and weather-induced biases [5]. Automatic algorithms and filters can correct obvious erroneous entries, e.g. outside typical dates of occurrence, observer variability or excessive bird numbers [5, 12, 13]. Other genuine entries artificially boosting bird numbers, such as multiple observations of the same birds (e.g. [13, 14]), survey data or exceptional movements caused by disturbance, remain in the database and require manual processing, though. This is especially relevant when dealing with absolute numbers. Accounts of handling and processing such data entries, particularly outside *eBird*, is sparse in literature.

In Europe, many bird portals are originally focussed on single quantitative observations (i.e. counts) and their qualitative content, not on checklists as *eBird*. The checklist feature is becoming more common also in Europe and its use is encouraged, especially as it relieves the problem of biased reporting through unknown presence or absence of common or unattractive species [2]. However, the checklist feature is missing in many current and most historical data entries which comprise already hundreds of millions of sightings, also retrospectively entered records covering about the last hundred years. Such historical data potentially represents a valuable treasure for studies in need of long-term data, e.g. related to climate change. It would thus be useful to gather experience with unstructured non-checklist (NCL) datasets and assess their features and challenges in data processing in a variety of research questions and also identify potential advantages over checklists, if any. Challenges and benefits of unstructured NCL datasets for breeding bird monitoring purposes have been investigated e.g. in Sweden [2] and in Denmark [15] based on the respective national bird portals. Movement research employing species occurrence (presence/absence) data from *eBird* checklists in the US gives a first idea of the potential of this data source in this field (e.g. [14, 16, 17]).

The few existing field ornithological publications derived from data from European bird portals, whose number of observations exceeds those of *eBird* in Europe, deal with small-scale questions related to distribution and occurrence during breeding or migration season [13, 18, 19]. Only the EuroBirdPortal website (www.eurobirdportal.org) shows large-scale migration progression and phenology derived from a mixed pool of bird data, including portals, at a very coarse resolution of eight zones for all of Europe. To the best of our knowledge there is no publication on large-scale bird movements based on NCL data only of any of the European bird repositories, and potential issues (or advantages) inherent to data analysis. On a global level, our understanding of broad-scale migrations on population level throughout the annual cycle are scarce [17]. So far migration phenology has been mainly described by data from birds either observed and/or ringed at specific bird observatories during determined periods of the year (e.g. [20–22]). NCL data could help fill knowledge gaps in this field of research for wider geographical areas and in the entire annual cycle. Especially in the context of climate change and its impact on migration timing and eventually population dynamics and survival, it is important to tap into new data sources to broaden our understanding of spatiotemporal migration dynamics.

In this article we test the suitability of NCL observations to obtain key figures of species-specific migration phenologies (onset, peak and end of migration, i.e. 5%, 50% and 95% percentiles, respectively), and migration progression in a latitudinal range of about 1000 km, and how these key figures compare to long-term ringing data. For this purpose, we analyse and process 10 years of NCL count data for all of Finland from the Finnish bird repository *Tiira* of BirdLife Finland and 40 years of autumn ringing data from the Hanko Bird Observatory. We hypothesise that unstructured NCL data is well suited to estimate the above-mentioned descriptors of migration for a broad range of species across large spatial scales and to identify latitudinal differences in migration timing, irrespective of inherent biases typically affecting breeding bird monitoring studies by NCL data. We further expect these descriptors to match well with ringing data for all common species regularly captured by standardized mist-netting schemes, i.e. mainly passerines. We discuss bird- and human-induced factors to be considered in handling NCL data from online portals and describe applied methodologies to address potential biases in the context of bird migration phenology.

## Material & methods

We analysed bird observations from the Finnish bird portal *Tiira* (https://www.tiira.fi/) for 115 common migrant species from 2010–2019 (see S1 Table). The database consisted of count data (without presence-absence features) pooled in 73 five-day periods, hereafter pentads, as introduced by [23] as a standard unit for migration studies, containing the species-specific sums of the respective bird numbers during the 10 selected years. The database consisted of all available observations of birds classified as migrating and stationary by observers, also potentially multiple entries of the same individuals, overall 400,749,314 birds. The reason for including both migratory and stationary birds was the difficulty of always telling birds’ behaviour unequivocally apart in the field. Our assumption was that once migration begins, bird numbers will steeply increase on top of the local numbers of breeding birds, indicating thus the onset of migration. This is particularly important also for quiet nocturnal migrants, whose actual migration otherwise remains unnoticed during the day. Similarly, it is often hard to tell whether some observation concerns the same or new birds, especially in case of common species and during migration.

**Fig 1:**
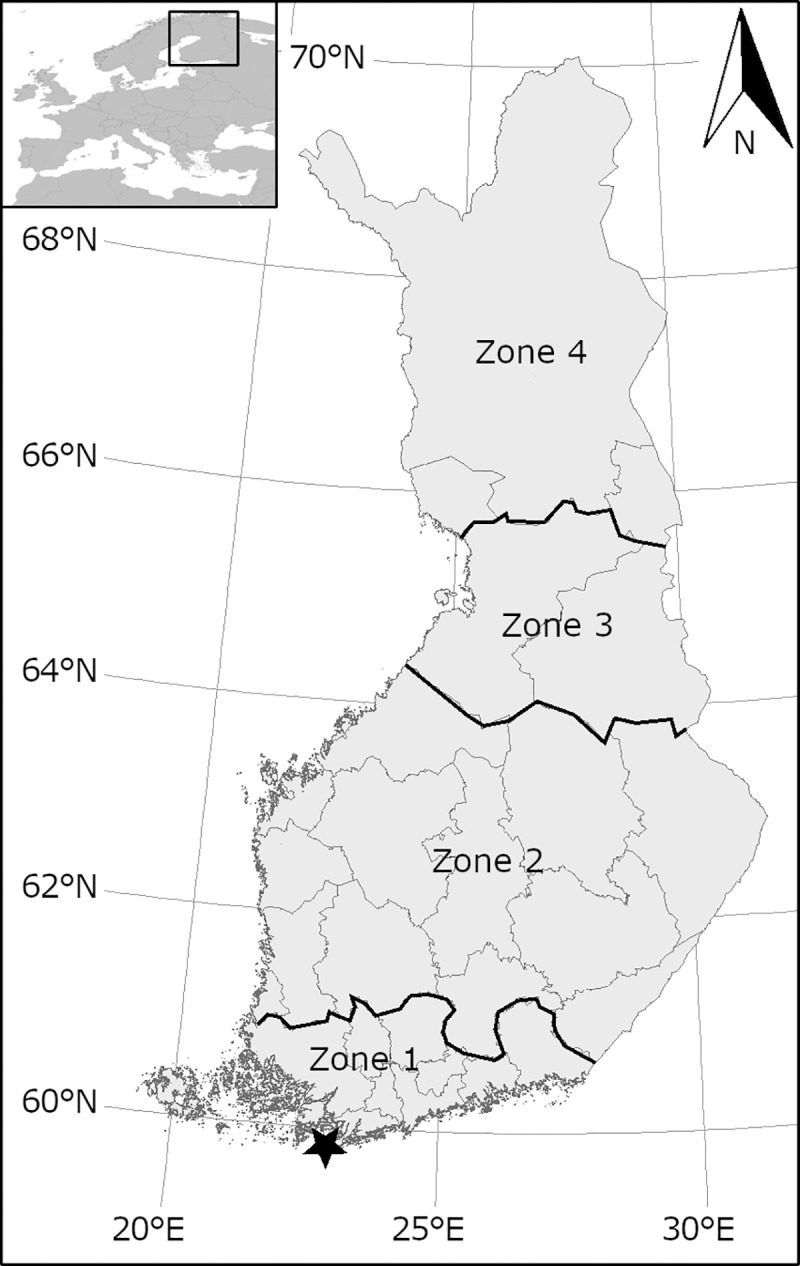
Map of Finland with the zones 1–4 used in the NCL data analysis and boundaries of 27 bird clubs. The star indicates the location of the Hanko Bird Observatory.

Data was pooled by the reporting area of the 27 regional bird clubs which were assigned to one of four regions on a North-South axis (Fig 1). This should enable the detection of latitudinal shifts in migration timing representative of each zone at an appropriate scale, while maintaining sufficiently large sample sizes for all species per area. The distances between the centres of neighbouring zones (about 250–300 km) agree well with the migration distances of about 200–400 km per flight bout of many bird species (e.g. [24, 25]). Depending on the scope of a study, the resolution could be also defined differently, i.e. coarsened or refined. Latitudinal shifts in migration timing might not be clearly discernible, though, if zones are too small. For example, for arctic migrants, such as geese, we also tested an alternative with partly longitudinal regions, as Siberian geese species could be expected to move on a SW-NE axis through Finland rather than on a N-S axis. However, occurrence patterns were similar, so we maintained the N-S regions for all species. Zones at the edge of or outside a species’ breeding range, as defined by the Finnish Breeding Bird Atlas [26], or regular migration flyway were excluded for the respective species. This concerns mostly the northern parts (zone 3 and 4) where species with rather southern distribution ranges (e.g. Eurasian blackbird *Turdus merula* and Blyth’s reed warbler *Acrocephalus dumetorum*) do not regularly occur, not even on migration. Through histograms we then gained an overview over the yearly occurrence and spring and autumn migration periods of all species. Based on our own field ornithological knowledge, we expected spring and autumn migration to show as either symmetrical or asymmetrical curves with typically one clear peak in each season, as opposed to very regular or irregular summer and winter presence without any distinct peaks. During this first inspection, we identified human- and bird-induced effects biasing the identification of migration periods which required adjustments during the data processing (see S1 Appendix).

### Data processing

Start and end of each migration period was visually determined as the pentad when bird numbers start to increase and form an outstanding peak for several pentads (more than three) after prior constant presence or absence, or start to taper off after a rapid decrease, respectively. Any non-migratory presence outside the identified migratory period was removed from the data, i.e. pentads were set to zero. To second start and end pentads of migration periods we consulted additionally existing literature on regional timing of Finnish migration periods (e.g. [27, 28]), if available. In some cases the procedure of determining clean migration curves was challenged by the human- and bird-induced effects described in S1 Appendix, so that the original NCL data had to be slightly modified by the following adjustments A1-A4. (A1) Extrapolation of existing unbiased pentad values to the zero level on either side of the true spring and autumn migration peaks (e.g. to cut and level out secondary peaks from breeding bird counts). We applied the following Eq (1) for extrapolation of 3–5 pentads in the total time series of the observed amounts of birds n in each pentad k:

$$A_{k}=n_{r}\;\left|k_{o}‐k\right|/\left|k_{o}‐k_{r}\right|$$ (1)

where A_k_ is the adjusted number of birds in pentad k; n_r_ is the observed number of birds in a reference pentad k_r_ of the same zone, where a clear trend in migration intensity can be observed; ko is the number of the first (if the adjustment is at the beginning of migration season) or last (if the adjustment is at the end of migration season) pentad where we choose to set A_ko_ = 0, i.e. no migration occurs. The extrapolation method is very simple, semi-linear and highly flexible.

(A2) Use of data from neighbouring zones if patterns were clearer there, e.g. start, peak and/or end of migration period were adjusted based on the respective timings in neighbouring zones and taking into account the interzonal migration speeds observed there (see also the example of Common redstart in S1 Appendix and the corresponding S1A Fig in S1 Appendix). In cases of severe data distortion, i.e. if migration peaks were absent (e.g as in Blyth’s reed warbler, S5A and S5B Fig in S1 Appendix), either part of or the entire migration period was replaced by migration phenologies from (A3) ringing and migration counts from the Hanko Bird Observatory (59°49’N, 22°54’E, https://haahka.halias.fi/) at the southwestern tip of Finland (Fig 1), or from (A4) Estonian waterbird migration counts (https://www.eoy.ee). A3 and A4 were used for zone 1 and 2 only. The adjustments A1-A4 concerned 39 (10%) of overall 382 species-zone combinations, specified in S1 Table.

After the adjustments, if any, we computed the running mean of three (rm3) or in highly irregular cases five (rm5) subsequent pentad counts of the respective season to smooth out short-term random fluctuations and highlight overall migration progression. For that purpose, three (or five) pentads were summed, i.e. (pentad_k-2_ +) pentad_k-1_ + pentad_k_ + pentad_k+1_ (+ pentad_k+2_), and divided by three (or five, respectively). In some cases, no smoothing was required in spring and/or autumn, as e.g. in the Dunlin (S4A and S4B Fig in S1 Appendix) which only required averaging over five pentads in autumn to even the irregular pattern. The start and end pentad of each species-specific migration period was chosen such that smoothing did not expand the duration of the respective migration period. The adjustment and smoothing procedures implemented for each species and zone are detailed in S1 Table with the respective keys A1-A4, rm3 and rm5, as well as the type of bias present. Overall, we aimed to conserve natural patterns, e.g. dynamics from different age groups or sexes as typically found in waders.

In a next step, the absolute count data of each pentad was converted into relative values (%) to facilitate comparison irrespective of absolute counts (see S1-S5 Figs in S1 Appendix). The 5%, 50% (median) and 95% percentile dates of spring and autumn migration were calculated for all species based on [29] to define the start, peak and end days of spring and autumn migration and to obtain figures comparable to literature findings (e.g. [30–33]). By doing so, the time unit of our analysis is days and not pentads. We found that smoothing time series may postpone or prepone median days by about one day. However, when checking for interannual variability of medians, annual medians varied by 0–14 days because of natural annual fluctuations in migration timing, so we considered the effect of smoothing as insignificant for median accuracy in the 10-year dataset. All analyses and visualizations were performed and generated in the program R [34].

### Statistical analysis

We used generalized linear mixed effects models with Gaussian error distribution to test for latitudinal migration progression in spring and autumn. The response variable was median migration date (50% percentile in Julian dates 1 Jan = 1) of species in a given zone and the analyses were conducted separately for spring and autumn migration. Our explanatory variables were zones (ordinal variable with four levels: zones 1–4) and species-specific mean spring or autumn median of all areas (continuous variable) as explanatory variables in the spring and autumn analysis, respectively. The latter was used to check for different dynamics in the progression of early and late migrant species through the zones. Both response variables were centred before the analyses. Species were included as random effects. We took the phylogeny of the species into account in the random structure of the model, as closely related species may have similar responses because of common ancestry. We downloaded a phylogeny set of the study species (source of trees: Ericson All Species: a set of 1000 trees with 9993 operational taxonomic units each) from www.birdtree.org [35]. The model included both main effects and interactions of the explanatory variables. The analysis was performed using the R package MCMCglmm [36]. We used 13000 iterations with thinning set to 10 in the analyses, i.e. the first 3000 iterations were discarded (burn-in period).

### Comparison of NCL data with ringing data

As an independent reference for our results, NCL medians of zone 1 were compared to medians derived from ringing data of the Hanko Bird Observatory in zone 1 from 1979–2019. Even though visual migration counts are executed alongside ringing at the observatory, we chose not to include this data, as it is partly entered into the *Tiira* bird portal and is hence part of the NCL archive. So, the ringing database was the most representative and comprehensive independent data available for the area both as to species and temporal range. 43 of 115 NCL species whose migration phenology was not replaced or supplemented by secondary data (i.e. potentially from Hanko) were included in this analysis. The selection included only landbirds, predominantly passerines, for which more than 50 ringing records were available (see S1 Table). Waders and raptors were excluded from the datasets as standardised mist-netting does not yield representative captures for these bird species. Following [31–33], the beginning of autumn migration season was set to 15 July, with some later dates for species breeding in the surroundings of the ringing station to reduce biases by resident birds (see ibid.). Like for the NCL data, ringing data was pooled in pentads and smoothed by running means of three pentads, i.e. by summing pentad_k-1_ + pentad_k_ + pentad_k+1_ and dividing their sum by three. To reduce outliers, we considered only species counts in the range of 5–95% of the migration season. To test for potential temporal shifts in medians through climate change, we conducted the analysis with data from 2000–2019 and 1979–2019. As we could not find better accordance with NCL data in the 20 years dataset, we used 40 years, especially also because this improved sample sizes considerably. We computed the Pearson correlation to check for correlations between number of captures and difference in median to detect potential biases originating from sample size. We generated boxplots for all species with 25 and 75 percentiles, medians and 5–95% limits, to compare temporal species occurrence in both data sources.

## Results

### NCL data

5%, 50% (medians) and 95% percentiles were obtained for 115 species in spring and autumn migration in Finland using NCL data (S1 Table). Smoothing or other processing was not required in 105 of 382 bird-zone combinations in spring (27%) and 36 of 382 combinations in autumn (9%). Smoothing by running means of three pentads only was required in 240 (63%) and 259 (68%) of 382 combinations in spring and autumn, respectively, and by running means of five pentads only in 18 (5%) and 67 (18%) of 382 combinations in spring and autumn, respectively. Migration patterns were adjusted by the methods A1-A4 in 19 bird-zone combinations in spring (5%, of which 14 and one required also smoothing by running mean of three and five pentads, respectively) in 10 species and in 20 combinations in autumn (5%, of which two and three combinations were smoothed by running mean of three and five pentads, respectively) in 13 species by using secondary data sources. A1, A2, A3 and A4 were applied 19, 1, 1 and 0 times in spring, respectively, and in autumn 4, 5, 12 and 3 times, respectively.

The model showed that spring migration medians were later with increasing latitude, but this effect weakened with the advancement of the season, i.e. the difference between latitudes was greatest in early spring migrants (Table 1). In autumn, medians were earlier in Northern regions, but there was no significant interaction between zones and mean migration timing and thus the migration progressed at a similar speed in early and late migratory species (Table 2).

**Table 1:** Parameter estimates (posterior mean including min-max values) and *p*-value based on the generalized linear mixed effects model explaining latitudinal migration progression in spring.

Variable	post.mean [min, max]	*p*-value
**Intercept**	124.07 [123.67, 124.49]	<0.001
**Zone**	3.87 [3.55, 4.26]	<0.001
**Spring median**	1.00 [0.98, 1.03]	<0.001
**Zone:Spring median**	-0.10 [-0.12, -0.07]	<0.001

Zone is zone 1–4 and spring median is the mean spring median date of the species.

**Table 2:** Parameter estimates (posterior mean including min-max values) and *p*-value based on the generalized linear mixed effects model explaining latitudinal migration progression in autumn.

Variable	post.mean [min, max]	*p*-value
**Intercept**	247.22 [246.70, 247.86]	<0.001
**Zone**	-2.47 [-2.99, -1.98]	<0.001
**Autumn median**	1.00 [0.98, 1.02]	<0.001
**Zone:Autumn median**	-0.01 [-0.03, 0.00]	0.138

Zone is zone 1–4 and autumn median is the mean autumn median date of species.

**Fig 2:**
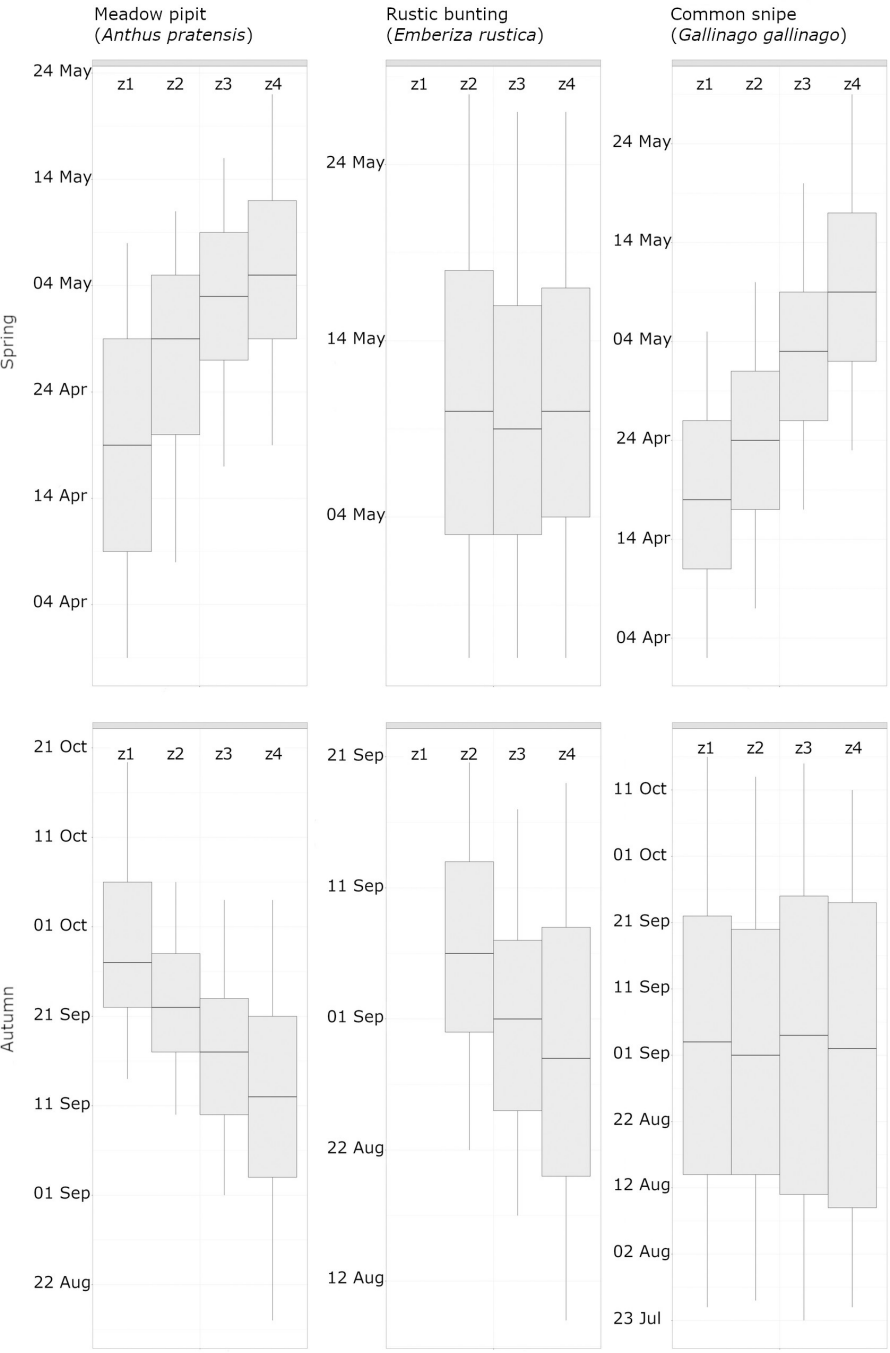
Progression of migration in spring and autumn across the zones 1–4. Meadow pipit *(Anthus Pratensis)*, Rustic bunting *(Emberiza rustica)* and Common snipe *(Gallinago gallinago)* are shown as examples of gradual vs. random progression in spring and autumn in the zones 1–4 (z1, z2, z3, z4). The boxplots consist of lower and upper limits at 5% and 95%, respectively, the plot hinges at 25% and 75%, and the median.

Generally, the progression of migration on a N-S axis was more pronounced in spring than in autumn (examples are shown in Fig 2).

In spring, only 14% (16 of 115 species) did not show a clear gradual progression from south to north. This concerned mainly waterfowl *(*Black-throated diver *(Gavia arctica)*, Red-throated diver *(Gavia stellata)*, Brent Goose *(Branta bernicla)*, Velvet scoter *(Melanitta fusca)*, Common scoter *(Melanitta nigra)*, Common eider *(Somateria mollissima)*) and waders (Dunlin *(Calidris alpina)*, Broad-billed sandpiper *(Calidris falcinellus)*, Curlew sandpiper *(Calidris ferruginea)*, Common ringed plover *(Charadrius hiaticula)*, Little stint *(Calidris minuta)*, Bar-tailed Godwit *(Limosa lapponica)*, Whimbrel *(Numenius arquata)*). Many of these are late spring migrants to Russian arctic and boreal breeding grounds and migration occurs more simultaneously in all areas. Non-gradual progression further occurred in one raptor (Common kestrel *(Falco tinnunculus)*), one non-passerine landbird (Common swift *(Apus apus)*) and one passerine species (Rustic bunting *(Emberiza rustica)*, Fig 2). In contrast in autumn, 70% (80 of 115) of the species did not exhibit a gradual progression from north to south. Gradual and non-gradual patterns were found in both passerines and non-passerines.

### Comparison ringing data vs. NCL data

**Fig 3:**
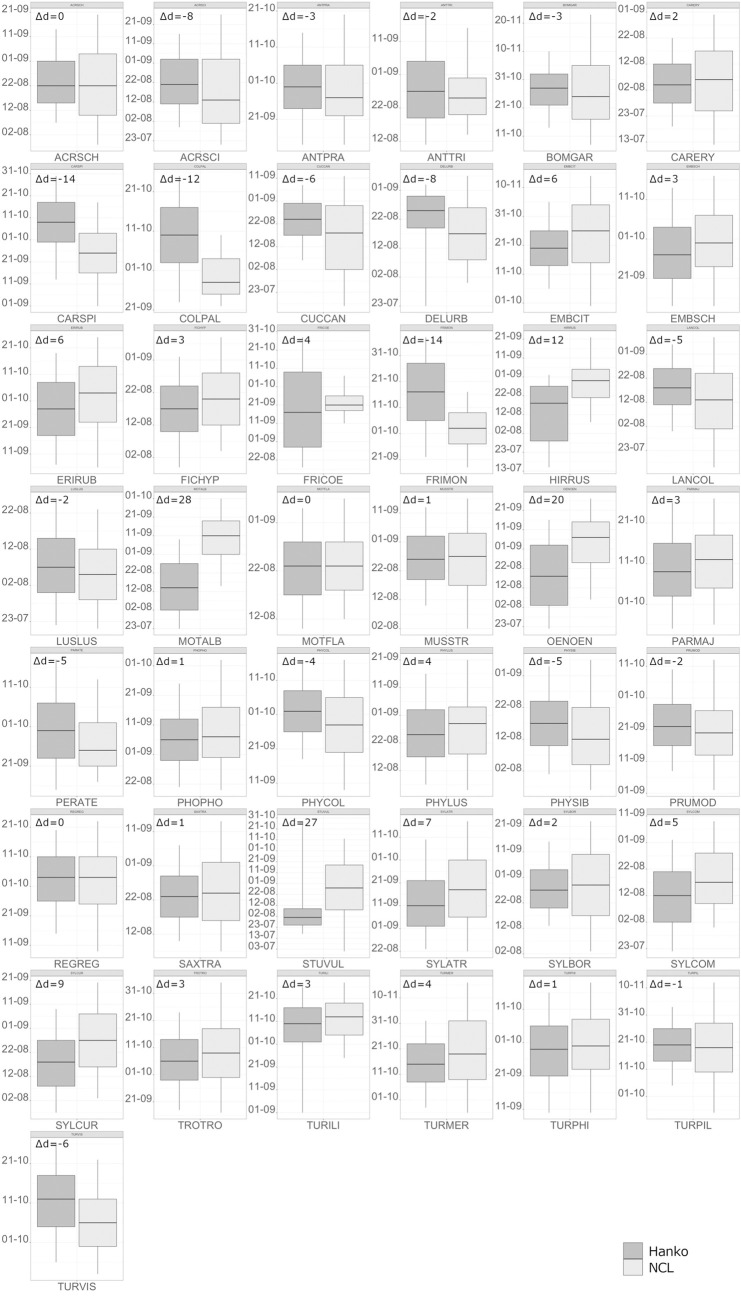
Comparison of medians from ringing (Hanko) and NCL data (NCL). Differences of medians between ringing (dark grey) and NCL data (light grey) are indicated as number of days (Δd). Negative differences mean the ringing median is later than the NCL median and vice versa for positive differences. For abbreviation keys see S1 Table.

Ringing and NCL medians differed between 0 and 28 days (Fig 3) with a mean difference of -0.64 days, i.e. the ringing median is later than the NCL median. 13 bird species exhibited a difference in median of |0–2| days, 11 of |3–4| days, 12 between |5–10| days and 7 between |11–28| days.

Largest differences were found in Wood pigeon *(Columbus palumbus)* (-12), White wagtail *(Motacilla alba)* (28), Barn swallow *(Hirundo rustica)* (12), Wheatear *(Oenanthe oenanthe)* (20), Brambling *(Fringilla montifringilla)* (-14), Siskin *(Carduelis spinus)* (-14) and Starling *(Sturnus vulgaris)* (27). We consider a difference of |0–4| days as equivalent medians (24 of 43 species) because of natural annual fluctuation of migration timing observed in the data, and in the discussion special attention will be paid to causes for differences >10 days. There was no correlation between sample size and difference in median (Pearson correlation coefficient r = -0.02), i.e. small two-digit sample sizes did not necessarily lead to large differences.

## Discussion

### NCL data

The analysis of the 10-year unstructured NCL dataset enabled the determination of species-specific migration phenology (start, median and end of migration) and latitudinal differences in migration timing for 115 bird species whose populations constitute the vast majority of the migration flow in four geographical regions in Finland. Phenological descriptors from ringing data largely supported the outcomes for a selection of the species. One benefit of the present methodology is that it is possible to estimate what proportion (percentiles) of each migrant species had already passed at any chosen day in the migration season. This is a clear step forward compared to available historical information in literature which typically just provided start and end dates of migration, rarely some medians and otherwise local examples of high migration counts, but no complete seasonal distributions (e.g. [27, 28, 37]). The percentiles further represent a more quantitative option in a relative sense compared to approaches based on presence-absence data from checklists (sensu [38]). Checklists provide probabilities of occurrence—also available from the present dataset—though no percentiles showing what proportion has migrated or is yet to pass. Even though these descriptors of migration phenology are basically already available from local bird observatory data (e.g. [26, 31–33]), another benefit of NCL data pools is the faster accumulation of a much higher number of observations with superior coverage. As to data processing, we estimate the effort of cleaning and preparing data to be comparable to data from standardized schemes of bird observatories based on methodologies described in literature (ibid).

The use of relative quantities helped obtain a more balanced view, also in cases of extreme differences between numbers of spring and autumn migrants. As to the use of pentads, it has been argued that pentads would falsify the progression and dynamics of migration, i.e. pentads may divide migration events into two different pentads [17, 30]. In this study, we find that there is a negligible effect of pentads on medians, if any, because we are dealing with median days, not median pentads. Furthermore, there is natural interannual variation in migration timing that may shift not only annual medians, but also start and end of migration by far more than some few days. So, we argue that the use of pentads did not significantly affect the representativeness of our results. Our approach could be also used with daily observations, but they might require some additional smoothing because of more daily fluctuations.

The detection, recognition, and handling of biases in NCL data require good field ornithological understanding to assess whether some issue was natural or not, as well as familiarity with relevant local to national human activities that affected the number of entries, e.g. surveys or hunting periods. Natural or real ecological variation in bird migration is due to environmental factors, i.e. weather conditions that cause fluctuations in bird numbers. These variations are also present in our data. However, we argue that “true” migration timing is an average over many years of variable annual timings influenced by environmental conditions. So, we believe, the 10-year dataset levelled out this environmental impact. A detailed analysis of weather impact on yearly migration patterns was beyond the scope of this study, though.

On the other hand, migration is probably observed imperfectly because of locally variable human- and bird-induced biases. Sampling effort is likely to vary in the course of the year, e.g. depending on weather conditions. The present analysis probably benefited overall from the fact that migration is a highly popular phenomenon attracting innumerous observers. Certain observers report very avidly practically every day, even in adverse weather conditions, and their presence or absence has a major impact on bird records. The risk of multiple entries of the same birds is particularly high at strongly frequented birding sites. However, we do not think that variability in human observation effort falsifies the massive numbers of migrating birds in such a systematic and persistent way that migration patterns (onset, peak and end) would disappear in our approach because sample sizes are typically very high (several thousands to millions of birds). Human observation effort and its variation can be assumed to be an approximately constant bias throughout a year and between years. We also expect that the pooling of 10 years of data reduces the impact of a potential observer effort bias and we think it did thus not bear any significance in our study, because we were looking for migration phenology (start, peak, end of migration) as *relative* distributions, rather than accurate counts. These biases can have a positive or negative impact on data quality, sometimes opposite to the impact on data for breeding bird monitoring. That is, for instance, observer preference for attractive or uncommon species leads to false absences undesired in breeding bird monitoring studies [2]. However, in the context of the present study, data of some common species benefited from this effect, as breeding records were few or absent and migration peaks better visible (e.g. as in the Blackbird, S3A Fig in S1 Appendix) because of lack of observers’ interest. At the same time, if such a common species is resident or partial migrant with a considerable proportion staying in the breeding area in winter, low avifaunal diversity (and thus boredom in birdwatchers) and many birds’ tendency to approach settlements (e.g. visits at feeders or flocking) increase winter records, which then can obscure the onset and end of migration, as e.g. in the Yellowhammer *(Emberiza citrinella)*. Species attractiveness has certainly a lower significance in spring migration, when people are still thirsty for the first arrival of both common and less common bird species and report them eagerly. More problematic than continuous biases is the presence of short-term events, such as surveys or hunting, which increase bird numbers in 1–3 subsequent pentads. In these cases, awareness and knowledge of local and national ornithological monitoring schemes and other events are necessary to separate human-induced increases from potential (unknown) natural phenomena. To avoid biases from surveys, it would be handy to be able to mark surveys by a dedicated field to be able to identify them as such and filter them out easily.

Contrary to the benefits observed in using bird checklists in breeding bird monitoring [2], the feature of mere presence/absence records, which is considered an improvement over unstructured quantitative NCL data, does not fully respond to the needs in migration studies, if quantitative count data is missing. Provided a species is an obligate migrant, presence/absence will then only indicate the beginning and end of the species’ presence in an area in the annual cycle, just like historical accounts in the pre-digital era (e.g. [28]), but no migration distributions over time (peaks or any fluctuations in between). In partial migrants or resident species, suitability of the data can be expected to be even lower. Ideally, observers should thus be encouraged to record numbers also on checklists whenever feasible and not retreat to the presence/absence feature. Admittedly, detectability and thus counting is strongly affected by a bird’s vocal activity [39], which is likely one of the reasons for the higher number of zones requiring adjustments in autumn. Similarly, staging and stopover behaviour affect detectability and observability. If species do not land, or travel at night, especially small birds are hard to detect, if they do not call. Waders may have enough fat reserves in spring to return to their breeding grounds without stopovers [40]. So, the spring migration occurrence in many wader species is shorter and less numerous than in autumn migration, which was also observed in the NCL database. To support adequate interpretation and data processing, it is reasonable to consult migration data collected by other methods, which might work better for certain species, such as ringing for elusive passerines (see below). Complementary data from ringing or in a future also from tagged birds could also help determine which populations pass a certain area, so that potential blurring or shifting of migration peaks through overlapping timing can be resolved.

Fluctuations and latitudinal differences in migration schedules, however, is mostly due to climatological or meteorological factors (e.g. vegetation, snow or ice cover) both at the breeding ground and en route [41], which are impossible to control for. Late or early onset of spring or winter or other unfavourable weather conditions can force birds to halt or accelerate departure or arrival, which again leads to more variation in the start, peak and end of migration [21]. This is probably the underlying reason for the clearer latitudinal effect observed by the model in spring, i.e. environmental conditions far north in early spring may slow down or speed up migration progression northwards. In autumn, however, the meteorological conditions seem less decisive, probably also because most birds leave already before conditions become really adverse. In any case, as observed in the dataset, annual start, peak and end of migration might vary as much as 1–2 weeks depending on environmental conditions. Our medians and migration periods are therefore to be considered average or climatological descriptors of migration phenology, rather than exact dates for a specific year.

Besides, the dominance of gradual migration progression from south to north in spring can be explained by the pressure to reach the breeding grounds as quickly as possible [42]. The cases of non-gradual progression concerned mostly waders and waterfowl. The reason for that could be that waders and waterfowl are enduring flyers which can easily cover long distances in one flight bout (e.g. [43]), highly likely across one or more zones established in this paper. Thus, migration advances more rapidly and the first birds appear at different latitudes practically simultaneously. A methodological bias can be excluded especially as the waders typically exhibit very nice observational data in each zone separately because of their high visibility at stopover sites. Another reason for non-gradual appearance in waterfowl could be a migration axis along SW-NE for the arctic populations, even though initial checking did not show any such effect. Similarly, species with an eastern migration route like Rustic bunting (Fig 2) would enter Finland on a broad north-south front from the east and thus the north-south approach of our zone system would not show major differences. However, this pattern was only evident in spring for Rustic Bunting and not observed in other migrants heading east, e.g. Bluethroat *(Luscinia svecica)* and Blyth’s reed warbler. In autumn, birds tend to linger longer [44], which results in a more heterogeneous migration progression. Variation in weather conditions, as described above, may contribute to the dominance of arbitrary patterns in autumn.

### Comparison Hanko ringing data vs. NCL data

It is well known that different surveying methods and equipment yield different outcomes of bird numbers and composition (e.g. [45–47]). Such is also the case when comparing ringing with visual and acoustic observations [48], and thus also in the present study with ringing and NCL data. Ringing data is, however, the only independent long-term database available in the area and it is important to become aware of its benefits and shortcomings to potentially complement NCL data.

In the present analysis, largest differences (>10 days) concerned mainly diurnal migrants (6 out of 7), while smaller or no differences were found in both nocturnal and diurnal long- and short-distance migrants. We suggest that the large differences result from varying habitat preferences, migratory and sedentary behaviour, age, size and arrival of wintering birds. The observatory is situated in coastal habitat, at the tip of an elongated peninsula, in a mixture of scrub and forest, with some reed beds as well as open and rocky shores. Species of open or semi-open landscapes such as the Barn swallow might fly by but will not settle in the vegetation and thus will not be caught. The same applies to Wheatear, which prefer rocky or open landscape, Brambling and Starling, which do form large flocks but stay in open areas. They nest only rarely in the surroundings of the observatory. Some species form large pre-migratory flocks or are otherwise gregarious, such as Starlings and Siskins, so high numbers do not necessarily indicate migration. Wood pigeon is typically easily observed, especially also in large flocks during autumn migration. Even though the highest daily migration counts at Hanko Bird Observatory exceed 10 000 birds every year, the Wood pigeon is only rarely captured by mist-netting because of its size and habits. Of 135 captured birds, only one was an adult. So captured individuals are most likely inexperienced juvenile birds which still linger in the forest or bushes and they do not reflect migration patterns of the main population. The ringed White Wagtails originated mostly from captures by wader cages, which were employed only irregularly for short time periods [32].

We rule out climate change as the main reason for median differences of more than 10 days, even though recent studies amongst others involving Finnish data point out to shifts in migration timing of about one week in the past 50 years [26]. Shifts stated in literature are much shorter than the observed large differences of up to 28 days and cannot fully explain the divergence. Also, when contrasting the outcomes of analysing 20 years (2000–2019) with 40 years of ringing data, we could not find any difference in patterns, i.e. the data from 20 years did not yield a closer match to the NCL medians than the 40-years dataset, rather the opposite, but that was most likely due to small sample sizes in the 20-years dataset.

So, overall, if we disregard the seven species with extreme differences in medians originating from capture biases in ringing, two thirds of the species (24 of 36) showed an agreement of 0–4 days in medians and one third (12 of 36) an agreement of 5–10 days. We interpret this outcome as support for the validity of the medians obtained from NCL data.

## Conclusions

Even though a minor proportion of species and zones was heavily affected by human- and bird-induced effects, overall processing effort was commensurate to the outcomes and data quality did not discourage the use of this data to describe migration phenologies. The model outcomes and ringing analysis support the credibility of the descriptors of migration phenology. We are confident that the basic concept of the present methodology can be applied to NCL data of all bird portals in phenological migration studies on species and population level, provided the availability of sufficient records (at best >100 per species per season) and as the case may be complementary local reference data for adjustments e.g. from ringing or literature. Our approach involved very basic information (bird species and numbers, and location—in our case pooled by bird clubs) of bird portal data, which is by default collected by any such platform. Only the reference data used for the adjustments would need to be chosen locally. Besides, knowledgeable field ornithologists are found in all European countries where comparable bird portals exist, which can be consulted for the ornithological interpretation of the data, if needed. Technical challenges are not to be expected as bird data can be readily handled by software commonly used for data analyses, such as R or similar. The broad spatial coverage of NCL data makes it an attractive complement to existing more sparsely scattered observational and ringing data from bird observatories. Even though the existence of online bird portals is still relatively short compared to ringing or observational archives of bird observatories, databases hold large amounts of data older than the portals, i.e. often more than 20 years. It could be worth trying out long-term analyses e.g. related to migration timing and climate change, for which currently data from standardized schemes is used. Besides, NCL data could be used as a complement by the growing community of radar ornithologists typically dealing with large-scale movements without access to species-specific information [49]. The data resolution of the present study fits well with weather radar density in Finland and can be adapted to other countries as required. We hope that the present study inspires more research based on NCL data.

## Supporting information

S1 Appendix The S1 Appendix details human- and bird-induced biases encountered and describes the data processing and respective adjustments in the example of the common redstart *(Phoenicurus phoenicurus)*. (PDF)

S1 Table The table contains the 115 species included in the study and respective percentiles, implemented processing and sample sizes in each zone. The information is presented in columns as follows: species: English and scientific species names and respective six-letter acronyms; zone: one of four zones used in the study region; Spr 5%, Spr 50%, Spr 95%: 5%, 50% and 95% percentiles for spring migration; Aut 5%, Aut 50%, Aut 95%: 5%, 50% and 95% percentiles for autumn migration; Proc Spr: adjustment procedures used in spring distributions; Proc Aut: adjustment procedures used in autumn distributions; n NCL Spr: sample sizes of spring; n NCL Aut: sample sizes of autumn of NCL data (and ringing data). Processing and adjustments in the respective columns contain the following abbreviations: rm3: running mean of three pentads; rm5: running mean of five pentads; adjustments, if any, implemented according to the five options (A1) linear extrapolation, (A2) neighbouring zones (A3) replacement by Hanko data (A4) replacement by Estonian waterbird migration counts; and underlying bird-induced (B) or human-induced (H) effects.
(PDF)
