# Supplementary material for: Challenges and benefits of using unstructured citizen science data to estimate seasonal timing of bird migration across large scales
Source: PLoS One. 2021 Feb 4;16(2):e0246572. doi: 10.1371/journal.pone.0246572 (PMC7861542; doi:10.1371/journal.pone.0246572)
Supplement: S1 Appendix — (PDF) [file pone.0246572.s001.pdf]

# S1 Appendix.

# Human- and bird-induced biases

In the following we detail the biases encountered during the inspection of the bird data and illustrate them and the respective processing procedure by specific examples.

## Human-induced effects

Multiple observations of the same birds by various observers at the same or nearby observation sites occurs especially in very popular and highly frequented areas along migration flyways. Another increase in bird observations is due to all kinds of survey results entered on the bird portal, such as winter line transects (pentads 1-6 Jan), "garden watch" (pentads 21-26 Jan) in wintertime or breeding bird line transects (pentads 5-10 Jun). Winter surveys do not affect migration patterns in this study. Biases from surveys concerns especially species common in Finland, which are otherwise not readily reported, e.g. the Common redstart *(Phoenicurus phoenicurus)* shows one true migration peak and a secondary one caused by breeding bird line transects in pentads 5-10 Jun in zones 2-4 (shown only zone 2 in S1 Fig A).

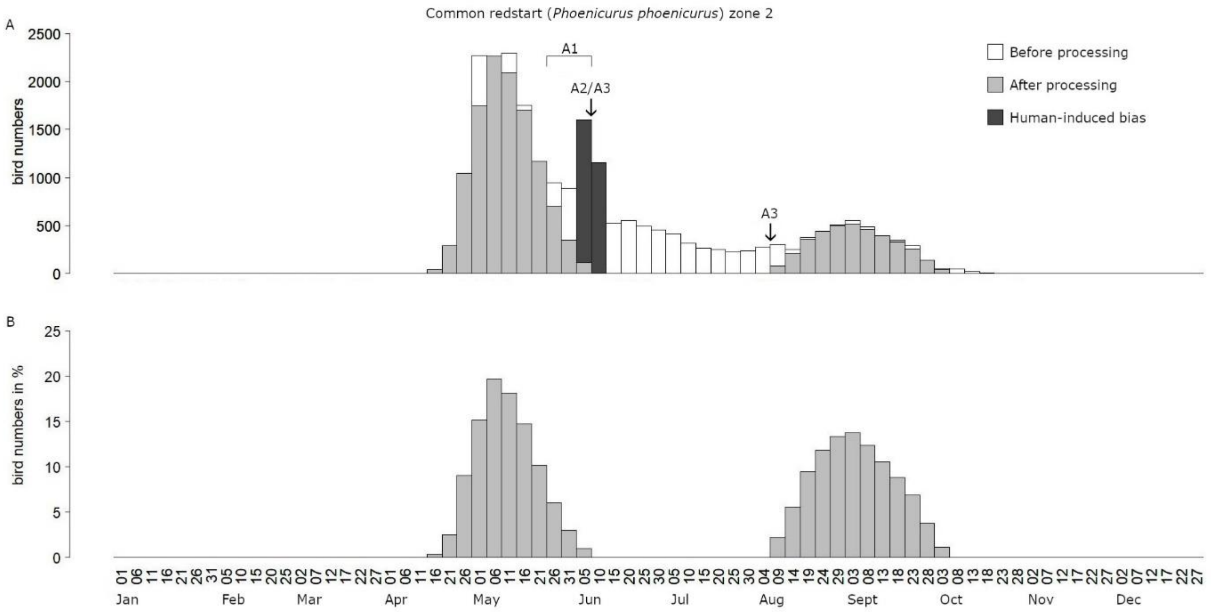


**S1 Fig. Annual occurrence of Common redstart *(Phoenicurus phoenicurus).*** NCL data set in zone 2 with bird numbers before and after data processing (A) and relative yearly migration distribution in percent (B) is shown. The x-axis shows the first day of each pentad. In S1 Fig A, the white bars show

the raw bird numbers, the light grey bars show bird numbers after adjustment A1 and smoothing, as well as end of spring migration and onset of autumn migration adopted from neighbouring zone 1 (A2) and the Hanko Bird Observatory (A3), with dark grey bars indicating removed human-induced biases from breeding bird counts.

A similar effect can be caused by unusually large numbers of ringing data entered on Tiira, especially in species whose reported numbers are otherwise relatively small, e.g. Sedge warbler (*Acrocephalus schoenobaenus*) after the singing period. Hunting, on the other hand, causes especially ducks to migrate locally and gather in protected areas, which can increase local numbers in mid to end of August, resembling moult migration but being spatiotemporally very concentrated (e.g. Teal *(Anas crecca)* in S2 Fig).

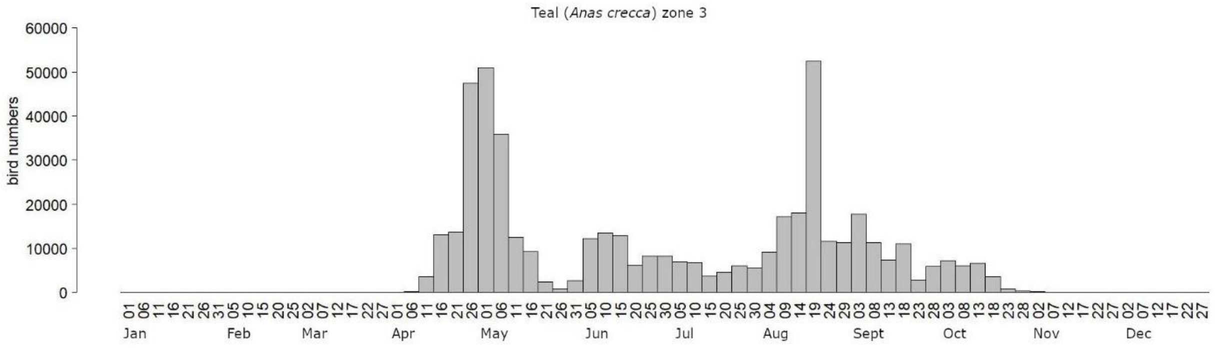


**S2 Fig. Annual occurrence of Teal *(Anas crecca).*** NCL data exhibits a major peak in pentad 19 Aug in zone 3 which is likely due to hunting and consequently accrual in protected areas near the city of Oulu. The x-axis shows the first day of each pentad.

Finally, species attractiveness and motivation to report especially common species varies with season. For instance, common resident or partial migrant species such as Eurasian blackbird in the zones 1-4 have practically no records from April to Sept, even though they are present in great numbers (S3 Fig A).

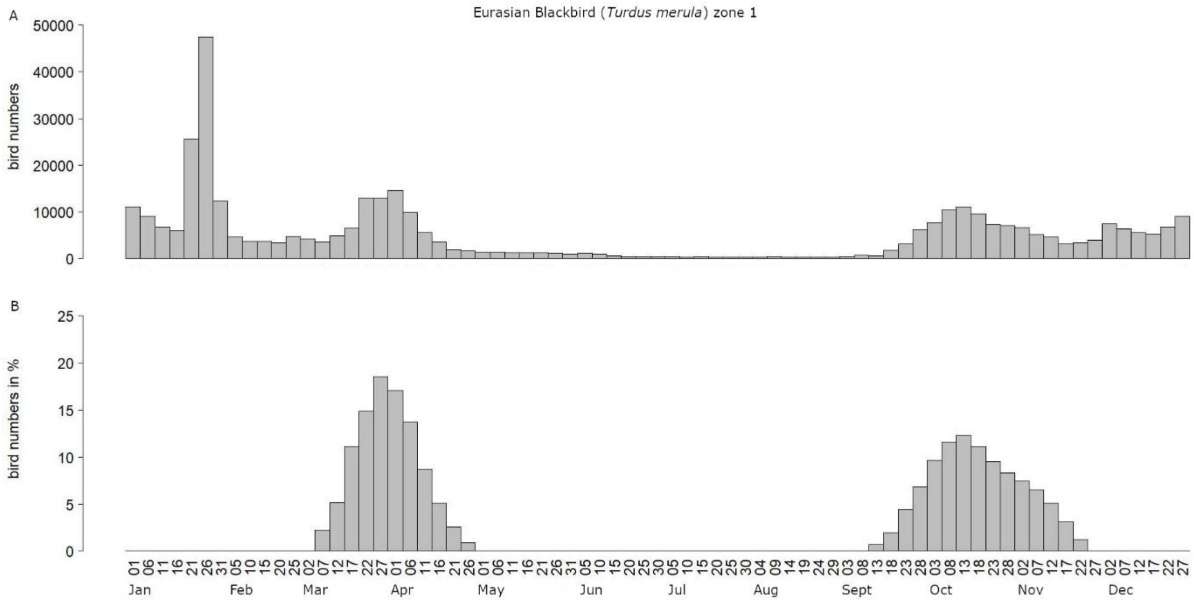


**S3 Fig. Annual occurrence of Eurasian blackbird *(Turdus merula)* in zone 1.** Before processing (A) with survey bias on 21-26 Jan, migration periods in spring (17 Mar-16 Apr) and autumn (18 Sep-17 Nov), and low summer occurrence through observer bias; and after processing (B). The x-axis shows the first day of each pentad.

# Bird-induced effects

In addition to migration periods, many species are present in the study area in summer and/or in winter. So, observation records of such resident birds may obscure the onset and/or end of migration, especially if paired with uneven or selective reporting by observers throughout the year. One such example is the Common Redstart where the end of spring migration and the onset of autumn migration are obscured by relatively large numbers of reported birds during summer (S1 Fig A). On the other hand, lack of data on breeding presence may actually facilitate the identification of migration periods, as in the Eurasian blackbird (S3 Fig A). Differences between spring and autumn migration strategies affect birds' observability, e.g. more time pressure in spring leads to less staging and fewer observations than in autumn. In addition, intraspecific differences in migration strategies (juveniles vs. adults, females vs. males) cause multiple or extended peaks in seasonal distributions. Such patterns are common in many wader species, such as Dunlin *(Calidris alpine)* (S4 Fig A).

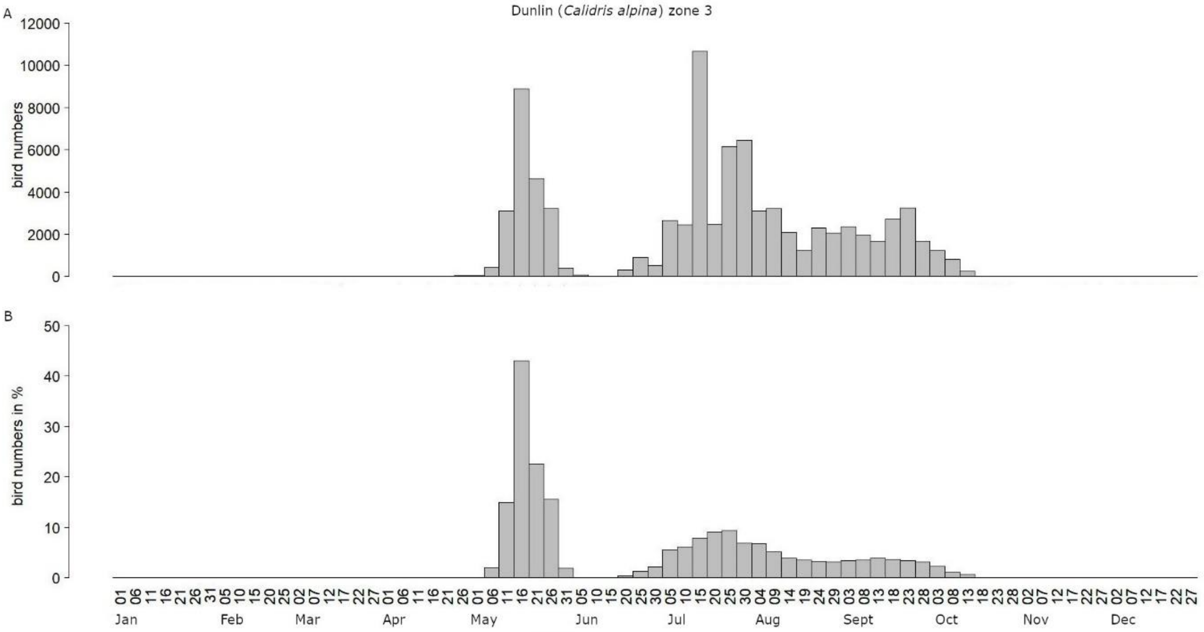


**S4 Fig. Annual occurrence of Dunlin *(Calidris alpine)* in zone 3.** An example of no adjustment in spring and running mean adjustment over five pentads in autumn before (A) and after (B) processing. The two peaks in the autumn season are due to different migration timing between age groups. The x-axis shows the first day of each pentad.

Also, the passage of different populations may have a similar effect. Species' observability depends further strongly on how vocal they are. Some species such as Blyth's reed warbler sing until they find

a mate in spring, but then become silent and elusive [1], and observations taper off without any autumn migration peak (S5 Fig A).

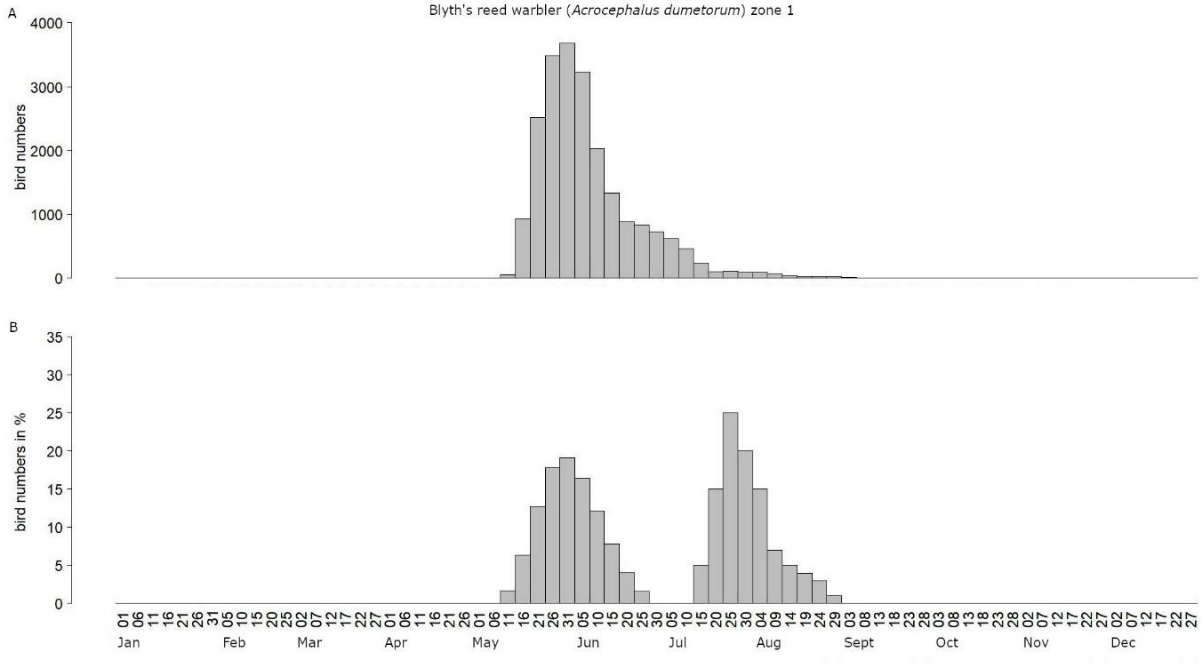


**S5 Fig. Annual occurrence of Blyth's reed warbler *(Acrocephalus dumetorum)* in zone 1**. Observations cease with advancement of the spring season because of the species' elusiveness (A). Autumn migration patterns (B) were derived from data from the Hanko Bird Observatory. The x-axis shows the first day of each pentad.

For species regularly calling during migration (e.g. Tree pipit *(Anthus trivialis)* or Dunnock *(Prunella modularis)*) or singing during stopover (e.g. Chiffchaff *(Phylloscopus collybita*)) clear migration peaks are also visible in autumn.

# Example of data processing and respective adjustments

We exemplify the adjustment and smoothing procedures by the challenging case shown in S1 Fig A and S1 Fig B of the Common redstart. In the original pentad data (S1 Fig A) the spring migration peak in zone 2 occurs during the pentads 1st to 11 May and bird numbers decrease from the pentad 11 May to 21 May. Subsequently, there is a second peak during the pentads 5 to 10 Jun that is not related to spring migration but to human-induced bias ("H" in S1 Table) from breeding bird counts. [2] suggests spring migration in zone 2 to end around the pentad 5 Jun, which is supported by the clear migration peak in zone 1 (corresponding to adjustment A2) and data from the Hanko Bird Observatory (A3). We estimate that the decrease in actual migration will continue after pentad 21 May, i.e. at pentad 10 Jun and thereafter no significant numbers of spring migrants arrive in zone 2. Accordingly, by selecting $k_r$ to be the value of pentad 21 May with $n_r$ approximately 1100 birds and $k_o$ to 5 Jun, the original bird numbers $n_k$ (1000, 900 and 1600) in S1 Fig A for the pentads k = 26 May, 31 May and 5 Jun, respectively, will be replaced with figures $A_k$ = 730, 370 and 0, respectively (corresponding to adjustment A1). In autumn, the start of migration was based on data from the Hanko Bird Observatory (i.e. A3) because it was masked by breeding presence. The final tweak for both seasons consisted in smoothing by running means of three pentads (rm3).

# References


1. Koskimies P. Breeding biology of Blyth's Reed Warbler *Acrocephalus dumetorum* in SE Finland. Ornis Fenn. 1980; 57: 26–32.
2. Haartman L von, Hildén O, Linkola P, Suomalainen P, Tenovuo R. Pohjolan linnut värikuvin I-II. Helsinki: Otava; 1963–1972.
