# Supplementary material for: Challenges and benefits of using unstructured citizen science data to estimate seasonal timing of bird migration across large scales
Source: PLoS One. 2021 Feb 4;16(2):e0246572. doi: 10.1371/journal.pone.0246572 (PMC7861542; doi:10.1371/journal.pone.0246572)
Supplement: S1 Table — The information is presented in columns as follows: species: English and scientific species names and respective six-letter acronyms; zone: one of four zones used in the study region; Spr 5%, Spr 50%, Spr 95%: 5%, 50% and 95% percentiles for spring migration; Aut 5%, Aut 50%, Aut 95%: 5%, 50% and 95% percentiles for autumn migration; Proc Spr: adjustment procedures used in spring distributions; Proc Aut: adjustment procedures used in autumn distributions; n NCL Spr: sample sizes of spring; n NCL Aut: sample sizes of autumn of NCL data (and ringing data). Processing and adjustments in the respective columns contain the following abbreviations: rm3: running mean of three pentads; rm5: running mean of five pentads; adjustments, if any, implemented according to the five options (A1) linear extrapolation, (A2) neighbouring zones (A3) replacement by Hanko data (A4) replacement by Estonian waterbird migration counts; and underlying bird-induced (B) or human-induced (H) effects. (PDF) [file pone.0246572.s002.pdf]

| Species | Zone | Spr 5% | Spr 50% | Spr 95% | Aut 5% | Aut 50% | Aut 95% | Proc Spr | Proc Aut | n NCL Spr | n NCL Aut (n Hanko) |
|---|---|---|---|---|---|---|---|---|---|---|---|
| Sparrowhawk (Accipiter nisus; ACCNIS) | 1 | 29 Mar | 16 Apr | 17 May | 21 Aug | 17 Sept | 19 Oct | rm3 | rm3 | 40706 | 357318 |
| | 2 | 01 Apr | 19 Apr | 17 May | 09 Aug | 16 Sept | 20 Oct | rm3 | rm3 | 33969 | 68544 |
| | 3 | 10 Apr | 26 Apr | 19 May | 11 Aug | 10 Sept | 13 Oct | rm3 | rm3 | 5881 | 6316 |
| | 4 | 10 Apr | 28 Apr | 21 May | 10 Aug | 05 Sept | 08 Oct | rm3 | rm3 | 2336 | 2500 |
| Blyth's reed warbler (Acrocephalus dumetorum; ACRDUM) | 1 | 19 May | 03 Jun | 21 Jun | 20 Jul | 31 Jul | 23 Aug | rm3 | A3(B) | 18079 | 818 |
| | 2 | 21 May | 05 Jun | 22 Jun | 20 Jul | 31 Jul | 16 Aug | rm3 | A3(B) | 40804 | 1550 |
| Marsh warbler (Acrocephalus palustris; ACRRIS) | 1 | 22 May | 08 Jun | 26 Jun | 20 Jul | 31 Jul | 21 Aug | rm3 | A3(B) | 9873 | 555 |
| | 2 | 24 May | 10 Jun | 28 Jun | 20 Jul | 31 Jul | 21 Aug | rm3 | A3(B) | 4793 | 205 |
| Sedge warbler (Acrocephalus schoenobaenus; ACRSCH) | 1 | 07 May | 26 May | 16 Jun | 29 Jul | 22 Aug | 20 Sep | rm3 | rm3 | 26085 | 15244 (799) |
| | 2 | 11 May | 30 May | 18 Jun | 01 Aug | 24 Aug | 17 Sep | rm3 | rm3 | 27069 | 6983 |
| | 3 | 16 May | 04 Jun | 22 Jun | 01 Aug | 23 Aug | 20 Sep | rm3 | A2(H) | 2425 | 1300 |
| | 4 | 22 May | 10 Jun | 02 Jul | 02 Aug | 21 Aug | 14 Sep | rm3 | rm3 | 1324 | 668 |
| Reed warbler (Acrocephalus scirpaceus; ACRSCI) | 1 | 09 May | 29 May | 21 Jun | 21 Jul | 13 Aug | 26 Sep | rm3 | rm3 | 7771 | 2745 (111) |
| | 2 | 13 May | 02 Jun | 22 Jun | 21 Jul | 11 Aug | 16 Sep | rm3 | rm3 | 2440 | 678 |
| Common sandpiper (Actitis hypoleucos; ACTHYP) | 1 | 22 Apr | 08 May | 27 May | 09 Jul | 01 Aug | 01 Sep | rm3 | rm3 | 19424 | 18530 |
| | 2 | 24 Apr | 09 May | 30 May | 03 Jul | 27 Jul | 28 Aug | rm3 | rm3 | 23868 | 19468 |
| | 3 | 01 May | 13 May | 28 May | 28 Jun | 23 Jul | 24 Aug | rm3 | rm3 | 3070 | 2695 |
| | 4 | 06 May | 19 May | 04 Jun | 27 Jun | 22 Jul | 23 Aug | rm3 | rm3 | 3023 | 3703 |
| Eurasian skylark (Alauda arvensis; ALAARV) | 1 | 10 Mar | 28 Mar | 15 Apr | 12 Sep | 28 Sept | 16 Oct | rm3 | rm3 | 449348 | 210573 |
| | 2 | 17 Mar | 04 Apr | 20 Apr | 11 Sep | 27 Sept | 12 Oct | rm3 | rm3 | 179224 | 71678 |
| | 3 | 26 Mar | 15 Apr | 03 May | 06 Sep | 27 Sept | 13 Oct | rm3 | rm3 | 8319 | 2472 |
| | 4 | 07 Apr | 20 Apr | 05 May | 07 Sep | 29 Sept | 16 Oct | rm3 | rm3 | 4513 | 541 |
| Northern pintail (Anas acuta; ANAACU) | 1 | 04 Apr | 21 Apr | 06 May | 18 Aug | 19 Sept | 21 Oct | none | rm5 | 67535 | 69650 |
| | 2 | 09 Apr | 24 Apr | 10 May | 13 Aug | 18 Sept | 12 Oct | none | rm5 | 91937 | 93090 |
| | 3 | 16 Apr | 29 Apr | 13 May | 30 Jul | 29 Aug | 13 Oct | none | rm5 | 42992 | 28639 |
| | 4 | 27 Apr | 09 May | 26 May | 16 Jul | 20 Aug | 30 Sep | none | rm5 | 6760 | 3296 |
| Northern shoveler (Anas clypeata; | 1 | 14 Apr | 01 May | 19 May | 05 Aug | 06 Sept | 12 Oct | rm3 | rm3 | 51178 | 60186 |
| | 2 | 18 Apr | 03 May | 22 May | 26 Jul | 06 Sept | 10 Oct | rm3 | rm3 | 36474 | 29047 |
| Teal (Anas crecca; ANACRE) | 1 | 04 Apr | 20 Apr | 04 May | 29 Jul | 03 Sept | 16 Oct | none | rm3 | 819392 | 663491 |
| | 2 | 11 Apr | 26 Apr | 08 May | 31 Jul | 29 Aug | 07 Oct | none | rm3 | 1074430 | 423986 |
| | 3 | 17 Apr | 03 May | 17 May | 01 Aug | 27 Aug | 11 Oct | rm3 | rm5 | 186398 | 207677 |
| | 4 | 27 Apr | 11 May | 26 May | 31 Jul | 28 Aug | 06 Oct | rm3 | rm3 | 43747 | 33760 |
| Wigeon (Anas penelope; ANAPEN) | 1 | 02 Apr | 20 Apr | 05 May | 02 Sep | 27 Sept | 26 Oct | none | rm3 | 269316 | 1341287 |
| | 2 | 12 Apr | 27 Apr | 10 May | 31 Aug | 24 Sept | 18 Oct | none | rm3 | 313961 | 819573 |
| | 3 | 16 Apr | 02 May | 16 May | 10 Aug | 22 Sept | 22 Oct | rm3 | rm3 | 53136 | 185314 |
| | 4 | 29 Apr | 12 May | 28 May | 17 Aug | 22 Sept | 16 Oct | rm3 | rm3 | 19547 | 178806 |
| Mallard (Anas platyrhynchos; ANAPLA) | 1 | 16 Mar | 07 Apr | 23 Apr | 06 Aug | 08 Oct | 25 Nov | rm3 | rm3 | 973293 | 1517144 |
| | 2 | 27 Mar | 14 Apr | 29 Apr | 06 Aug | 29 Sept | 13 Nov | rm3 | rm3 | 908489 | 1263188 |
| | 3 | 03 Apr | 19 Apr | 05 May | 12 Aug | 06 Oct | 15 Nov | rm3 | rm5 | 74844 | 193113 |
| | 4 | 13 Apr | 29 Apr | 15 May | 20 Aug | 07 Oct | 01 Nov | rm3 | rm3 | 35655 | 85175 |
| Greater white-fronted goose (Anser albifrons; ANSALB) | 1 | 28 Mar | 23 Apr | 19 May | 26 Sep | 09 Oct | 23 Oct | rm3 | rm3 | 983723 | 506076 |
| | 2 | 03 Apr | 26 Apr | 15 May | 27 Sep | 10 Oct | 21 Oct | rm3 | rm3 | 514102 | 847347 |
| Grey-lag goose (Anser anser; ANSANS) | 1 | 16 Mar | 03 Apr | 20 Apr | 24 Jul | 31 Aug | 28 Sep | rm3 | rm3 | 307644 | 2574291 |
| | 2 | 20 Mar | 07 Apr | 23 Apr | 18 Jul | 15 Aug | 15 Sep | rm3 | rm3 | 409474 | 539320 |
| | 3 | 31 Mar | 15 Apr | 29 Apr | 02 Jul | 31 Jul | 24 Aug | rm3 | rm3 | 85151 | 167732 |

| Species | Zone | Spr 5% | Spr 50% | Spr 95% | Aut 5% | Aut 50% | Aut 95% | Proc Spr | Proc Aut | n NCL Spr | n NCL Aut (n Hanko) |
|---|---|---|---|---|---|---|---|---|---|---|---|
| Taiga bean goose (Anser fabalis fabalis; ANSFABFAB) | 1 | 11 Mar | 01 Apr | 24 Apr | 08 Sep | 29 Sept | 19 Oct | rm3 | rm3 | 179572 | 8541 |
| | 2 | 11 Mar | 02 Apr | 24 Apr | 07 Sep | 27 Sept | 16 Oct | rm3 | rm3 | 652822 | 20754 |
| | 3 | 04 Apr | 21 Apr | 05 May | 03 Sep | 27 Sept | 21 Oct | rm3 | rm3 | 127862 | 29100 |
| | 4 | 19 Apr | 03 May | 17 May | 19 Aug | 10 Sept | 05 Oct | rm3 | rm5 | 6519 | 866 |
| Tundra bean goose (Anser fabalis rossicus; ANSFABROS) | 1 | 29 Mar | 20 Apr | 11 May | 24 Sep | 09 Oct | 25 Oct | rm3 | rm3 | 740351 | 113139 |
| | 2 | 31 Mar | 24 Apr | 12 May | 20 Sep | 05 Oct | 22 Oct | rm3 | rm3 | 576025 | 96663 |
| Meadow pipit (Anthus pratensis; ANTPRA) | 1 | 30 Mar | 19 Apr | 08 May | 14 Sep | 27 Sept | 20 Oct | rm3 | none | 58364 | 303115 (184) |
| | 2 | 08 Apr | 29 Apr | 11 May | 10 Sep | 22 Sept | 06 Oct | none | none | 106897 | 464318 |
| | 3 | 17 Apr | 03 May | 16 May | 01 Sep | 17 Sept | 04 Oct | rm3 | rm3 | 12736 | 33355 |
| | 4 | 19 Apr | 05 May | 22 May | 18 Aug | 12 Sept | 04 Oct | rm3 | rm3 | 6612 | 17479 |
| Tree pipit (Anthus trivialis; ANTTRI) | 1 | 22 Apr | 05 May | 21 May | 14 Aug | 25 Aug | 15 Sep | rm3 | none | 21218 | 411851 (971) |
| | 2 | 23 Apr | 06 May | 22 May | 11 Aug | 24 Aug | 15 Sep | rm3 | none | 23474 | 134487 |
| | 3 | 27 Apr | 10 May | 29 May | 12 Aug | 25 Aug | 17 Sep | rm5 | rm3 | 5250 | 7157 |
| | 4 | 29 Apr | 16 May | 05 Jun | 03 Aug | 24 Aug | 18 Sep | rm3 | rm3 | 955 | 1494 |
| Common swift (Apus apus; APUAPU) | 1 | 12 May | 29 May | 14 Jun | 29 Jul | 10 Aug | 28 Aug | rm3 | rm3 | 299808 | 235392 |
| | 2 | 13 May | 30 May | 14 Jun | 28 Jul | 09 Aug | 26 Aug | rm3 | rm3 | 176000 | 104977 |
| | 3 | 16 May | 29 May | 16 Jun | 27 Jul | 09 Aug | 25 Aug | rm3 | rm3 | 18116 | 9051 |
| Tufted duck (Aythya fuligula; AYTFUL) | 1 | 12 Mar | 12 Apr | 11 May | 20 Sep | 25 Oct | 20 Nov | rm3 | rm3 | 1040217 | 959413 |
| | 2 | 21 Mar | 27 Apr | 16 May | 12 Sep | 15 Oct | 09 Nov | rm3 | rm3 | 696367 | 296984 |
| | 3 | 23 Apr | 07 May | 21 May | 06 Sep | 05 Oct | 31 Oct | rm3 | rm3 | 110866 | 61098 |
| | 4 | 01 May | 14 May | 29 May | 23 Aug | 21 Sept | 15 Oct | rm3 | rm3 | 50537 | 43438 |
| Waxwing (Bombycilla garrulus; BOMGAR) | 1 | 12 Mar | 02 Apr | 06 May | 08 Oct | 25 Oct | 23 Nov | rm5 | rm5 | 75934 | 1171455 (2630) |
| | 2 | 13 Mar | 08 Apr | 12 May | 30 Sep | 18 Oct | 17 Nov | rm5 | rm5 | 208852 | 1330394 |
| | 3 | 20 Mar | 25 Apr | 19 May | 26 Sep | 11 Oct | 09 Nov | rm5 | rm5 | 34594 | 181298 |
| | 4 | 22 Mar | 18 Apr | 24 May | 21 Sep | 12 Oct | 12 Nov | rm5 | rm5 | 14143 | 259770 |
| Brent goose (Branta bernicla; BRABER) | 1 | 21 May | 25 May | 01 Jun | 13 Sep | 27 Sept | 13 Oct | none | rm3 | 8741514 | 438944 |
| | 2 | 21 May | 25 May | 02 Jun | 12 Sep | 26 Sept | 10 Oct | none | rm3 | 191503 | 1276536 |
| Canada goose (Branta canadensis; BRACAN) | 1 | 14 Mar | 31 Mar | 18 Apr | 15 Oct | 07 Nov | 04 Dec | rm3 | rm3 | 265671 | 496368 |
| | 2 | 19 Mar | 08 Apr | 27 Apr | 13 Oct | 01 Nov | 30 Nov | rm3 | rm3 | 84639 | 240067 |
| Barnacle goose (Branta leucopsis; BRALEU) | 1 | 11 May | 18 May | 25 May | 23 Sep | 05 Oct | 21 Oct | none | none | 18854237 | 34294745 |
| | 2 | 11 May | 19 May | 26 May | 19 Sep | 01 Oct | 19 Oct | none | none | 8351167 | 44487376 |
| | 3 | 11 May | 19 May | 28 May | 17 Sep | 29 Sept | 17 Oct | none | rm3 | 15300 | 124673 |
| Goldeneye (Bucephala clangula; BUCCLA) | 1 | 9 Mar | 31 Mar | 26 Apr | 04 Sep | 13 Oct | 22 Nov | rm3 | rm5 | 506736 | 1157381 |
| | 2 | 23 Mar | 19 Apr | 13 May | 31 Aug | 01 Oct | 05 Nov | rm3 | rm5 | 637270 | 1508528 |
| | 3 | 08 Apr | 01 May | 21 May | 03 Sep | 11 Oct | 11 Nov | rm3 | rm5 | 86142 | 83558 |
| | 4 | 13 Apr | 04 May | 24 May | 23 Aug | 28 Sept | 26 Oct | rm3 | rm5 | 71163 | 49099 |
| Common buzzard (Buteo buteo; BUTBUT) | 1 | 17 Mar | 09 Apr | 16 May | 30 Aug | 28 Sept | 19 Oct | rm3 | rm3 | 29243 | 108339 |
| | 2 | 22 Mar | 14 Apr | 14 May | 12 Aug | 09 Sept | 03 Oct | rm3 | rm3 | 36179 | 36169 |
| | 3 | 08 Apr | 24 Apr | 20 May | 19 Aug | 09 Sept | 26 Sep | rm3 | rm3 | 2260 | 1961 |
| Rough-legged buzzard (Buteo lagopus; BUTLAG) | 1 | 05 Apr | 15 Apr | 01 May | 19 Sep | 06 Oct | 27 Oct | none | rm3 | 11440 | 21224 |
| | 2 | 10 Apr | 20 Apr | 03 May | 17 Sep | 02 Oct | 22 Oct | none | rm3 | 31529 | 17657 |
| | 3 | 16 Apr | 25 Apr | 07 May | 16 Sep | 02 Oct | 17 Oct | none | rm3 | 17399 | 5517 |
| | 4 | 16 Apr | 27 Apr | 11 May | 04 Sep | 28 Sept | 13 Oct | none | rm3 | 5377 | 3571 |
| Sanderling (Calidris alba; CALALB) | 1 | 12 May | 28 May | 11 Jun | 21 Jul | 20 Aug | 30 Sep | rm5 | rm5 | 277 | 2376 |
| | 2 | 18 May | 03 Jun | 18 Jun | 22 Jul | 20 Aug | 02 Oct | rm5 | rm5 | 379 | 4644 |
| | 3 | 19 May | 04 Jun | 21 Jun | 20 Jul | 29 Aug | 05 Oct | rm5 | rm5 | 88 | 2604 |

| Species | Zone | Spr 5% | Spr 50% | Spr 95% | Aut 5% | Aut 50% | Aut 95% | Proc Spr | Proc Aut | n NCL Spr | n NCL Aut (n Hanko) |
|---|---|---|---|---|---|---|---|---|---|---|---|
| Dunlin (Calidris alpina; CALALP) | 1 | 17 May | 26 May | 31 May | 08 Jul | 02 Aug | 22 Sep | none | rm3 | 292452 | 270309 |
| | 2 | 12 May | 22 May | 01 Jun | 06 Jul | 31 Jul | 25 Sep | none | rm5 | 50628 | 412446 |
| | 3 | 12 May | 20 May | 30 May | 06 Jul | 05 Aug | 02 Oct | none | rm5 | 20625 | 61831 |
| Red knot (Calidris canuta; CALCAN) | 1 | 22 May | 29 May | 07 Jun | 21 Jul | 08 Aug | 02 Sep | none | rm3 | 105655 | 10203 |
| | 2 | 19 May | 31 May | 11 Jun | 17 Jul | 07 Aug | 09 Sep | rm3 | rm3 | 6358 | 23677 |
| Broad-billed sandpiper (Calidris falcinellus; CALFAL) | 1 | 22 May | 29 May | 05 Jun | 09 Jul | 25 Jul | 23 Aug | none | rm3 | 13606 | 1670 |
| | 2 | 23 May | 29 May | 05 Jun | 11 Jul | 08 Aug | 26 Aug | none | rm3 | 17315 | 1696 |
| | 3 | 23 May | 29 May | 07 Jun | 15 Jul | 05 Aug | 20 Aug | none | rm3 | 7212 | 308 |
| | 4 | 22 May | 01 Jun | 16 Jun | 07 Jul | 28 Jul | 18 Aug | rm3 | A2(B,H) | 1289 | 181 |
| Curlew sandpiper (Calidris ferruginea; CALFER) | 1 | 07 May | 22 May | 05 Jun | 08 Jul | 30 Jul | 02 Sep | rm3 | rm3 | 276 | 12701 |
| | 2 | 16 May | 29 May | 10 Jun | 08 Jul | 25 Jul | 01 Sep | rm3 | rm3 | 300 | 18544 |
| | 3 | 13 May | 25 May | 11 Jun | 07 Jul | 28 Jul | 05 Sep | rm3 | rm5 | 54 | 1930 |
| Little stint (Calidris minuta; CALMIN) | 1 | 17 May | 28 May | 09 Jun | 20 Jul | 28 Aug | 25 Sep | none | rm3 | 730 | 6772 |
| | 2 | 17 May | 29 May | 12 Jun | 20 Jul | 25 Aug | 17 Sep | rm3 | rm3 | 1732 | 17105 |
| | 3 | 17 May | 29 May | 10 Jun | 23 Jul | 25 Aug | 14 Sep | rm3 | rm3 | 279 | 4608 |
| Ruff (Calidris pugnax; CALPUG) | 1 | 29 Apr | 08 May | 19 May | 10 Aug | 04 Sept | 24 Sep | none | none | 330646 | 379119 |
| | 2 | 02 May | 12 May | 20 May | 05 Aug | 30 Aug | 18 Sep | none | none | 919297 | 273701 |
| | 3 | 07 May | 14 May | 23 May | 25 Jul | 28 Aug | 20 Sep | none | rm3 | 146651 | 74374 |
| | 4 | 08 May | 16 May | 31 May | 30 Jul | 25 Aug | 19 Sep | none | rm3 | 50915 | 5339 |
| Linnet (Carduelis cannabina; CARCAN) | 1 | 20 Mar | 04 Apr | 03 May | 14 Sep | 30 Sept | 20 Oct | rm3 | rm3 | 53624 | 101405 |
| | 2 | 23 Mar | 09 Apr | 05 May | 13 Sep | 28 Sept | 18 Oct | rm3 | rm3 | 23773 | 21985 |
| Common rosefinch (Carpodacus erythrinus; CARERY) | 1 | 14 May | 25 May | 11 Jun | 12 Jul | 06 Aug | 31 Aug | none | rm3 | 18840 | 4164 (71) |
| | 2 | 15 May | 26 May | 12 Jun | 11 Jul | 01 Aug | 26 Aug | none | rm3 | 20120 | 3041 |
| | 3 | 14 May | 27 May | 13 Jun | 05 Jul | 21 Jul | 12 Aug | rm3 | rm3 | 2565 | 230 |
| Eurasian siskin (Carduelis spinus; CARSPI) | 1 | 26 Feb | 10 Apr | 11 May | 01 Sep | 25 Sept | 18 Oct | rm5 | rm3 | 112711 | 1891333 (5265) |
| | 2 | 15 Mar | 23 Apr | 24 May | 26 Aug | 20 Sept | 11 Oct | rm5 | rm3 | 297668 | 819544 |
| | 3 | 29 Mar | 26 Apr | 25 May | 23 Aug | 14 Sept | 08 Oct | rm5 | rm5 | 25024 | 42264 |
| | 4 | 22 Mar | 26 Apr | 26 May | 12 Aug | 11 Sept | 02 Oct | rm5 | rm5 | 12195 | 24431 |
| Common ringed plover (Charadrius hiaticula; CHAHIA) | 1 | 07 May | 24 May | 12 Jun | 02 Aug | 20 Aug | 14 Sep | rm3 | rm3 | 43818 | 102451 |
| | 2 | 07 May | 22 May | 11 Jun | 30 Jul | 19 Aug | 16 Sep | rm3 | rm3 | 90420 | 166477 |
| | 3 | 07 May | 21 May | 10 Jun | 22 Jul | 12 Aug | 13 Sep | rm3 | rm3 | 11282 | 25410 |
| Long-tailed duck (Clangula hyemalis; CLAHYE) | 1 | 04 May | 17 May | 24 May | 28 Sep | 15 Oct | 07 Nov | none | rm3 | 16821531 | 8156979 |
| | 2 | 08 May | 18 May | 26 May | 25 Sep | 10 Oct | 25 Oct | none | rm5 | 638193 | 662193 |
| | 3 | 10 May | 19 May | 31 May | 29 Sep | 17 Oct | 15 Nov | rm3 | rm5 | 30182 | 5129 |
| Wood pigeon (Columba palumbus; COLPAL) | 1 | 27 Mar | 10 Apr | 27 Apr | 22 Sep | 28 Sept | 10 Oct | rm3 | none | 921602 | 4590664 (117) |
| | 2 | 31 Mar | 13 Apr | 01 May | 10 Sep | 26 Sept | 07 Oct | none | none | 1273704 | 1966912 |
| | 3 | 04 Apr | 18 Apr | 06 May | 29 Aug | 21 Sept | 03 Oct | rm3 | rm3 | 151192 | 42264 |
| | 4 | 07 Apr | 22 Apr | 12 May | 29 Aug | 20 Sept | 05 Oct | rm3 | rm3 | 43530 | 17224 |
| Hooded crow (Corvus cornix; CORNIX) | 1 | 26 Feb | 21 Mar | 19 Apr | 02 Oct | 16 Oct | 01 Nov | rm3 | none | 194546 | 613633 |
| | 2 | 28 Feb | 24 Mar | 20 Apr | 01 Oct | 13 Oct | 02 Nov | rm3 | none | 245926 | 457274 |
| | 3 | 4 Mar | 30 Mar | 02 May | 19 Sep | 06 Oct | 27 Oct | rm3 | rm3 | 29916 | 17804 |
| | 4 | 14 Mar | 09 Apr | 06 May | 04 Sep | 30 Sept | 27 Oct | rm5 | rm5 | 14184 | 29109 |
| Jackdaw (Corvus monedula; CORMON) | 1 | 3 Mar | 26 Mar | 18 Apr | 03 Oct | 15 Oct | 01 Nov | rm3 | none | 389169 | 2253750 |
| | 2 | 4 Mar | 29 Mar | 20 Apr | 28 Sep | 11 Oct | 29 Oct | rm3 | none | 777151 | 1373021 |
| | 3 | 3 Mar | 02 Apr | 29 Apr | 14 Sep | 05 Oct | 27 Oct | rm5 | rm5 | 47880 | 36659 |

| Species | Zone | Spr 5% | Spr 50% | Spr 95% | Aut 5% | Aut 50% | Aut 95% | Proc Spr | Proc Aut | n NCL Spr | n NCL Aut (n Hanko) |
|---|---|---|---|---|---|---|---|---|---|---|---|
| Cuckoo (Cuculus canorus; CUCCAN) | 1 | 03 May | 18 May | 05 Jun | 17 Jul | 18 Aug | 12 Sep | A1(B),rm3 | rm3 | 13514 | 3194 (119) |
| | 2 | 04 May | 20 May | 10 Jun | 19 Jul | 16 Aug | 09 Sep | A1(B),rm3 | rm3 | 22021 | 2608 |
| | 3 | 09 May | 23 May | 10 Jun | 28 Jul | 16 Aug | 04 Sep | A1(B),rm3 | rm3 | 1748 | 350 |
| | 4 | 13 May | 27 May | 12 Jun | 26 Jul | 15 Aug | 07 Sep | A1(B),rm3 | rm3 | 1622 | 185 |
| Blue tit (Cyanistes caeruleus; CYACAE) | 1 | - | - | - | 20 Sep | 11 Oct | 06 Nov | rm3 | rm3 | - | 432825 |
| Whooper swan (Cygnus cygnus; CYGCYG) | 1 | 11 Mar | 02 Apr | 26 Apr | 28 Sep | 07 Nov | 22 Dec | rm3 | rm3 | 775636 | 973432 |
| | 2 | 13 Mar | 06 Apr | 27 Apr | 26 Sep | 29 Oct | 06 Dec | rm3 | rm3 | 1861219 | 2515069 |
| | 3 | 19 Mar | 15 Apr | 06 May | 24 Sep | 20 Oct | 16 Nov | rm3 | rm3 | 326757 | 523636 |
| | 4 | 06 Apr | 09 May | 11 Jun | 17 Sep | 11 Oct | 06 Nov | rm3 | rm3 | 114426 | 480169 |
| House martin (Delichon urbica; DELURB) | 1 | 27 Apr | 14 May | 03 Jun | 31 Jul | 17 Aug | 06 Sep | rm3 | rm3 | 58688 | 67587 (156) |
| | 2 | 29 Apr | 16 May | 07 Jun | 28 Jul | 17 Aug | 03 Sep | rm3 | rm3 | 59680 | 78856 |
| | 3 | 07 May | 25 May | 17 Jun | 25 Jul | 16 Aug | 05 Sep | rm3 | rm3 | 8591 | 8483 |
| | 4 | 09 May | 31 May | 19 Jun | 31 Jul | 13 Aug | 01 Sep | rm5 | rm3 | 5951 | 11762 |
| Yellowhammer (Emberiza citrinella; EMBCIT) | 1 | 15 Mar | 01 Apr | 01 May | 30 Sep | 26 Oct | 14 Nov | rm5 | rm5 | 41468 | 100374 (85) |
| | 2 | 20 Mar | 06 Apr | 05 May | 25 Sep | 25 Oct | 09 Nov | rm5 | rm5 | 45013 | 100690 |
| | 3 | 22 Mar | 07 Apr | 06 May | 19 Sep | 14 Oct | 03 Nov | rm3 | rm5 | 12214 | 6310 |
| Rustic bunting (Emberiza rustica; EMBRUS) | 2 | 26 Apr | 10 May | 28 May | 22 Aug | 06 Sept | 21 Sep | rm3 | rm3 | 736 | 2800 |
| | 3 | 26 Apr | 09 May | 27 May | 17 Aug | 01 Sept | 17 Sep | rm3 | rm3 | 920 | 1358 |
| | 4 | 26 Apr | 10 May | 27 May | 09 Aug | 29 Aug | 19 Sep | rm3 | rm3 | 730 | 2159 |
| Reed bunting (Emberiza schoeniclus; EMBSCH) | 1 | 26 Mar | 14 Apr | 12 May | 14 Sep | 30 Sept | 17 Oct | rm3 | none | 23834 | 51290 (327) |
| | 2 | 01 Apr | 21 Apr | 19 May | 14 Sep | 24 Sept | 11 Oct | rm3 | none | 31022 | 48814 |
| | 3 | 13 Apr | 01 May | 22 May | 30 Aug | 20 Sept | 07 Oct | rm3 | none | 4451 | 9953 |
| | 4 | 21 Apr | 09 May | 01 Jun | 11 Aug | 09 Sept | 05 Oct | rm3 | rm3 | 4178 | 10400 |
| European robin (Erithacus rubecula; ERIRUB) | 1 | 02 Apr | 22 Apr | 11 May | 06 Sep | 04 Oct | 25 Oct | rm3 | rm3 | 132069 | 121020 (13145) |
| | 2 | 02 Apr | 19 Apr | 16 May | 28 Aug | 27 Sept | 22 Oct | rm3 | rm3 | 28772 | 51826 |
| | 3 | 08 Apr | 26 Apr | 19 May | 21 Aug | 28 Sept | 20 Oct | rm3 | rm3 | 2820 | 4292 |
| | 4 | 11 Apr | 28 Apr | 21 May | 21 Aug | 26 Sept | 19 Oct | rm3 | rm3 | 1757 | 2504 |
| Common kestrel (Falco tinnunculus; FALTIN) | 1 | 02 Apr | 23 Apr | 25 May | 15 Aug | 07 Sept | 29 Sep | rm3 | rm3 | 18564 | 45254 |
| | 2 | 29 Mar | 19 Apr | 25 May | 13 Aug | 06 Sept | 30 Sep | rm3 | rm3 | 35519 | 35051 |
| | 3 | 09 Apr | 27 Apr | 28 May | 11 Aug | 02 Sept | 29 Sep | rm3 | rm3 | 5596 | 2550 |
| | 4 | 14 Apr | 05 May | 01 Jun | 04 Aug | 24 Aug | 20 Sep | rm3 | rm3 | 3381 | 1728 |
| Pied flycatcher (Ficedula hypoleuca; FICHYP) | 1 | 26 Apr | 12 May | 29 May | 04 Aug | 20 Aug | 08 Sep | rm3 | rm3 | 24822 | 7298 (1674) |
| | 2 | 27 Apr | 11 May | 29 May | 26 Jul | 13 Aug | 04 Sep | rm3 | rm3 | 17989 | 3124 |
| | 3 | 02 May | 15 May | 03 Jun | 21 Jul | 12 Aug | 01 Sep | rm3 | rm3 | 2457 | 270 |
| | 4 | 03 May | 17 May | 04 Jun | 22 Jul | 14 Aug | 02 Sep | rm3 | rm3 | 2375 | 367 |
| Common chaffinch (Fringilla coelebs; FRICOE) | 1 | 28 Mar | 12 Apr | 26 Apr | 11 Sep | 21 Sept | 07 Oct | rm3 | none | 1390626 | 6612177 (2449) |
| | 2 | 03 Apr | 15 Apr | 01 May | 28 Aug | 17 Sept | 02 Oct | none | rm3 | 2743751 | 4807751 |
| | 3 | 07 Apr | 22 Apr | 04 May | 23 Aug | 13 Sept | 04 Oct | rm3 | rm3 | 182218 | 89146 |
| | 4 | 08 Apr | 23 Apr | 11 May | 19 Aug | 13 Sept | 06 Oct | rm3 | rm3 | 48413 | 50764 |
| Brambling (Fringilla montifringilla; FRIMON) | 1 | 08 Apr | 21 Apr | 04 May | 18 Sep | 03 Oct | 17 Oct | rm3 | rm3 | 62151 | 850363 (567) |
| | 2 | 17 Apr | 28 Apr | 09 May | 09 Sep | 27 Sept | 12 Oct | none | rm3 | 399258 | 943300 |
| | 3 | 22 Apr | 04 May | 15 May | 14 Aug | 15 Sept | 09 Oct | rm3 | rm5 | 117392 | 85668 |
| | 4 | 25 Apr | 08 May | 24 May | 11 Aug | 18 Sept | 11 Oct | rm3 | rm5 | 69906 | 48019 |

| Species | Zone | Spr 5% | Spr 50% | Spr 95% | Aut 5% | Aut 50% | Aut 95% | Proc Spr | Proc Aut | n NCL Spr | n NCL Aut (n Hanko) |
|---|---|---|---|---|---|---|---|---|---|---|---|
| Common snipe (Gallinago gallinago; GALGAL) | 1 | 02 Apr | 18 Apr | 05 May | 25 Jul | 03 Sept | 16 Oct | rm3 | rm5 | 69733 | 119668 |
| | 2 | 07 Apr | 24 Apr | 10 May | 26 Jul | 01 Sept | 13 Oct | rm3 | rm3 | 74151 | 72164 |
| | 3 | 17 Apr | 03 May | 20 May | 23 Jul | 04 Sept | 15 Oct | rm3 | rm5 | 6442 | 6772 |
| | 4 | 23 Apr | 09 May | 29 May | 25 Jul | 02 Sept | 11 Oct | rm3 | rm3 | 3665 | 1838 |
| Black-throated diver (Gavia arctica; GAVARC) | 1 | 25 Apr | 19 May | 30 May | 23 Aug | 23 Sept | 12 Oct | none | rm3 | 636426 | 41868 |
| | 2 | 22 Apr | 14 May | 01 Jun | 17 Aug | 19 Sept | 14 Oct | rm3 | rm5 | 276241 | 91934 |
| | 3 | 29 Apr | 15 May | 01 Jun | 08 Aug | 11 Sept | 09 Oct | rm3 | rm3 | 20689 | 3887 |
| | 4 | 06 May | 22 May | 16 Jun | 01 Aug | 03 Sept | 02 Oct | rm3 | rm3 | 10260 | 6334 |
| Red-throated diver (Gavia stellata; GAVSTE) | 1 | 16 Apr | 16 May | 05 Jun | 15 Sep | 05 Oct | 18 Nov | rm3 | rm5 | 47071 | 8426 |
| | 2 | 13 Apr | 02 May | 29 May | 11 Sep | 29 Sept | 13 Nov | rm3 | rm5 | 93315 | 27823 |
| | 3 | 26 Apr | 15 May | 05 Jun | 18 Sep | 03 Oct | 27 Oct | rm3 | rm3 | 8561 | 290 |
| Common crane (Grus grus; GRUGRU) | 1 | 02 Apr | 15 Apr | 26 Apr | 04 Sep | 21 Sept | 05 Oct | none | rm3 | 1329396 | 3414956 |
| | 2 | 05 Apr | 18 Apr | 04 May | 29 Aug | 20 Sept | 04 Oct | none | rm3 | 1023532 | 5227117 |
| | 3 | 13 Apr | 24 Apr | 08 May | 22 Aug | 13 Sept | 30 Sep | none | rm3 | 284435 | 710225 |
| | 4 | 19 Apr | 28 Apr | 14 May | 21 Aug | 13 Sept | 30 Sep | none | rm3 | 55604 | 129378 |
| Oystercatcher (Haematopus ostralegus; HAEOST) | 1 | 12 Apr | 01 May | 08 May | 15 Jul | 03 Aug | 28 Aug | none | rm3 | 283967 | 25469 |
| Icterine warbler (Hippolais icterina; HIPICT) | 1 | 13 May | 29 May | 17 Jun | 19 Jul | 03 Aug | 22 Aug | none | A3(B) | 16686 | 1202 |
| | 2 | 15 May | 31 May | 18 Jun | 19 Jul | 03 Aug | 22 Aug | rm3 | A3(B) | 5960 | 295 |
| Barn swallow (Hirundo rustica; HIRRUS) | 1 | 01 May | 14 May | 03 Jun | 08 Aug | 30 Aug | 22 Sep | none | none | 173152 | 1171911 (178) |
| | 2 | 30 Apr | 15 May | 05 Jun | 02 Aug | 26 Aug | 16 Sep | rm3 | none | 179354 | 753419 |
| | 3 | 06 May | 22 May | 07 Jun | 04 Aug | 26 Aug | 19 Sep | rm3 | rm3 | 18310 | 51884 |
| | 4 | 07 May | 23 May | 12 Jun | 26 Jul | 20 Aug | 19 Sep | rm3 | rm3 | 6548 | 17363 |
| Red-backed shrike (Lanius collurio; LANCOL) | 1 | 10 May | 26 May | 09 Jun | 16 Jul | 13 Aug | 08 Sep | rm3 | rm3 | 10623 | 28957 (465) |
| | 2 | 11 May | 31 May | 18 Jun | 10 Jul | 05 Aug | 03 Sep | rm3 | rm3 | 5763 | 18541 |
| Herring gull (Larus argentatus; LARARG) | 1 | 5 Mar | 26 Mar | 16 Apr | 09 Aug | 12 Oct | 25 Nov | rm3 | rm5 | 401821 | 1078494 |
| | 2 | 8 Mar | 30 Mar | 18 Apr | 10 Aug | 11 Oct | 26 Nov | rm3 | rm5 | 1653300 | 2240381 |
| | 3 | 12 Mar | 05 Apr | 24 Apr | 23 Aug | 12 Oct | 25 Nov | rm3 | rm5 | 272232 | 393508 |
| | 4 | 31 Mar | 18 Apr | 06 May | 21 Aug | 15 Oct | 21 Nov | rm3 | rm5 | 102912 | 187494 |
| Common gull (Larus canus; LARCAN) | 1 | 3 Apr | 22 Apr | 11 May | 29 Jul | 3 Oct | 18 Nov | rm3 | rm3 | 612505 | 1090658 |
| | 2 | 7 Apr | 22 Apr | 11 May | 28 Jul | 23 Sept | 9 Nov | rm3 | rm5 | 534453 | 1912499 |
| | 3 | 11 Apr | 28 Apr | 18 May | 22 Jul | 28 Sept | 7 Nov | rm3 | rm5 | 30284 | 38196 |
| | 4 | 17 Apr | 3 May | 22 May | 6 Jul | 18 Aug | 19 Oct | rm3 | rm5 | 19855 | 6311 |
| Lesser black-backed gull (Larus fuscus; LARFUS) | 1 | 02 Apr | 18 Apr | 05 May | 11 Jul | 10 Aug | 21 Sep | rm3 | rm5 | 29818 | 35721 |
| | 2 | 05 Apr | 18 Apr | 03 May | 29 Jun | 02 Aug | 09 Sep | rm3 | rm5 | 102334 | 74306 |
| | 3 | 10 Apr | 24 Apr | 09 May | 28 Jun | 07 Aug | 13 Oct | rm3 | rm5 | 4243 | 1502 |
| | 4 | 19 Apr | 03 May | 16 May | 28 Jun | 02 Aug | 08 Sep | rm3 | rm5 | 14244 | 3215 |
| Black-headed gull (Larus ridibundus; LARRID) | 1 | 30 Mar | 14 Apr | 02 May | 08 Jul | 26 Jul | 16 Aug | rm3 | A1,3(B, H) | 1781045 | 218671 |
| | 2 | 05 Apr | 18 Apr | 06 May | 03 Jul | 21 Jul | 11 Aug | rm3 | A1,3(B, H) | 4342578 | 726938 |
| | 3 | 11 Apr | 22 Apr | 06 May | 28 Jun | 16 Jul | 06 Aug | rm3 | A1,3(B, H) | 438317 | 35151 |
| | 4 | 16 Apr | 30 Apr | 20 May | 23 Jun | 11 Jul | 01 Aug | rm3 | A1,3(B, H) | 290979 | 27074 |
| Bar-tailed godwit (Limosa lapponica; LIMLAP) | 1 | 06 May | 21 May | 01 Jun | 17 Jul | 04 Aug | 29 Aug | none | rm3 | 141176 | 16889 |
| | 2 | 19 Apr | 08 May | 22 May | 15 Jul | 30 Jul | 25 Aug | rm3 | rm3 | 70130 | 32538 |
| Thrush nightingale (Luscinia luscinia; LUSLUS) | 1 | 07 May | 23 May | 09 Jun | 21 Jul | 05 Aug | 23 Aug | rm3 | rm3 | 36381 | 1106 (177) |
| | 2 | 10 May | 27 May | 10 Jun | 20 Jul | 03 Aug | 21 Aug | rm3 | rm3 | 26046 | 298 |

| Species | Zone | Spr 5% | Spr 50% | Spr 95% | Aut 5% | Aut 50% | Aut 95% | Proc Spr | Proc Aut | n NCL Spr | n NCL Aut (n Hanko) |
|---|---|---|---|---|---|---|---|---|---|---|---|
| Bluethroat (Luscinia svecica; LUSSVE) | 1 | 07 May | 15 May | 24 May | 28 Aug | 10 Sept | 29 Sep | none | none | 3660 | 3013 |
| | 2 | 07 May | 16 May | 24 May | 29 Aug | 10 Sept | 27 Sep | none | none | 3357 | 3312 |
| | 3 | 07 May | 18 May | 28 May | 24 Aug | 06 Sept | 23 Sep | rm3 | rm3 | 1169 | 868 |
| | 4 | 10 May | 23 May | 06 Jun | 17 Aug | 02 Sept | 18 Sep | rm3 | rm3 | 1699 | 5147 |
| Velvet scoter (Melanitta fusca; MELFUS) | 1 | 18 Apr | 14 May | 03 Jun | 29 Jul | 01 Oct | 31 Oct | none | A4(B),rm5 | 584881 | 135830 |
| | 2 | 19 Apr | 17 May | 27 May | 17 Jul | 29 Sept | 22 Nov | none | rm5 | 706488 | 111322 |
| | 3 | 13 May | 19 May | 26 May | 17 Jul | 24 Oct | 01 Dec | none | rm5 | 66793 | 5184 |
| Common scoter (Melanitta nigra; MELNIG) | 1 | 03 May | 14 May | 21 May | 07 Jul | 01 Aug | 17 Sep | none | A4(B),rm5 | 14004258 | 1760249 |
| | 2 | 11 Apr | 08 May | 26 May | 19 Jul | 14 Sept | 11 Nov | rm3 | rm5 | 1998493 | 594371 |
| | 3 | 08 May | 19 May | 26 May | 20 Jul | 15 Sept | 10 Nov | none | A2(B) | 205934 | 27727 |
| Goosander (Mergus merganser; MERMER) | 1 | 19 Mar | 16 Apr | 07 May | 18 Nov | 21 Sept | 1 Jan | rm3 | rm5 | 880382 | 1210805 |
| | 2 | 28 Mar | 23 Apr | 25 May | 29 Sep | 08 Nov | 15 Dec | rm3 | rm3 | 410333 | 2204466 |
| | 3 | 19 Apr | 09 May | 28 May | 17 Sep | 16 Oct | 11 Nov | rm3 | rm3 | 63381 | 101856 |
| | 4 | 21 Apr | 17 May | 05 Jun | 09 Sep | 04 Oct | 25 Oct | rm3 | rm3 | 26121 | 261560 |
| Red-breasted merganser (Mergus serrator; MERSER) | 1 | 24 Mar | 02 May | 26 May | 11 Sep | 08 Oct | 05 Nov | rm3 | rm3 | 137411 | 38779 |
| | 2 | 11 Apr | 02 May | 27 May | 25 Aug | 25 Sept | 20 Oct | rm3 | A4(B),rm5 | 185111 | 96774 |
| | 3 | 27 Apr | 11 May | 29 May | 17 Aug | 20 Sept | 19 Oct | rm3 | rm5 | 12189 | 4968 |
| | 4 | 05 May | 20 May | 09 Jun | 22 Aug | 26 Sept | 25 Oct | A1(B),rm5 | rm5 | 5040 | 5014 |
| White wagtail (Motacilla alba; MOTALB) | 1 | 03 Apr | 19 Apr | 09 May | 15 Aug | 11 Sept | 01 Oct | none | none | 122983 | 360664 (420) |
| | 2 | 08 Apr | 20 Apr | 05 May | 07 Aug | 05 Sept | 27 Sep | none | rm3 | 165093 | 275935 |
| | 3 | 12 Apr | 25 Apr | 12 May | 08 Aug | 02 Sept | 27 Sep | rm3 | rm3 | 21619 | 17438 |
| | 4 | 13 Apr | 28 Apr | 15 May | 05 Aug | 28 Aug | 27 Sep | rm3 | rm3 | 13616 | 14740 |
| Yellow wagtail (Motacilla flava; MOTFLA) | 1 | 03 May | 14 May | 29 May | 12 Aug | 23 Aug | 06 Sep | none | none | 29582 | 273329 (336) |
| | 2 | 05 May | 14 May | 24 May | 11 Aug | 23 Aug | 06 Sep | none | none | 65778 | 195618 |
| | 3 | 08 May | 18 May | 28 May | 01 Aug | 17 Aug | 02 Sep | none | rm3 | 15944 | 21710 |
| | 4 | 08 May | 21 May | 03 Jun | 29 Jul | 13 Aug | 01 Sep | rm3 | rm3 | 7364 | 11119 |
| Spotted flycatcher (Muscicapa striata; MUSSTR) | 1 | 11 May | 24 May | 09 Jun | 02 Aug | 27 Aug | 16 Sep | none | none | 25844 | 26805 (1323) |
| | 2 | 11 May | 28 May | 14 Jun | 29 Jul | 23 Aug | 17 Sep | rm3 | rm3 | 16194 | 13746 |
| | 3 | 12 May | 26 May | 14 Jun | 26 Jul | 24 Aug | 18 Sep | A1(H),rm3 | rm5 | 1133 | 1518 |
| | 4 | 14 May | 29 May | 16 Jun | 19 Jul | 16 Aug | 11 Sep | A1(H),rm3 | rm3 | 806 | 1290 |
| Curlew (Numenius arquata; NUMARQ) | 1 | 12 Apr | 21 Apr | 30 Apr | 27 Jul | 11 Aug | 27 Aug | none | rm3 | 256475 | 7631 |
| | 2 | 12 Apr | 20 Apr | 29 Apr | 21 Jul | 07 Aug | 25 Aug | none | rm3 | 315178 | 16406 |
| | 3 | 17 Apr | 24 Apr | 05 May | 20 Jul | 05 Aug | 21 Aug | none | rm3 | 46954 | 2269 |
| | 4 | 21 Apr | 27 Apr | 09 May | 18 Jul | 07 Aug | 23 Aug | none | rm3 | 27050 | 1389 |
| Whimbrel (Numenius phaeopus; NUMPHA) | 1 | 27 Apr | 07 May | 17 May | 27 Jun | 21 Jul | 12 Aug | none | rm3 | 36250 | 19111 |
| | 2 | 26 Apr | 08 May | 20 May | 22 Jun | 20 Jul | 13 Aug | none | rm3 | 22106 | 12451 |
| | 3 | 27 Apr | 10 May | 28 May | 16 Jun | 18 Jul | 14 Aug | none | rm5 | 3181 | 2517 |
| | 4 | 02 May | 13 May | 27 May | 14 Jun | 18 Jul | 14 Aug | none | A2(B) | 2361 | 2317 |
| Wheatear (Oenanthe oenanthe; OENOEN) | 1 | 13 Apr | 06 May | 27 May | 06 Aug | 07 Sept | 27 Sep | rm3 | none | 31563 | 37121 (141) |
| | 2 | 17 Apr | 09 May | 24 May | 10 Aug | 07 Sept | 26 Sep | rm3 | none | 22857 | 34156 |
| | 3 | 22 Apr | 11 May | 27 May | 06 Aug | 06 Sept | 27 Sep | rm3 | rm3 | 3070 | 1876 |
| | 4 | 27 Apr | 19 May | 07 Jun | 30 Jul | 27 Aug | 25 Sep | rm3 | rm3 | 2564 | 1683 |
| Great tit (Parus major; PARMAJ) | 1 | - | - | - | 26 Sep | 12 Oct | 27 Oct | rm3 | rm3 | - | 311165 (43368) |
| Honey buzzard (Pernis apivorus; PERAPI) | 1 | 13 May | 23 May | 08 Jun | 23 Aug | 03 Sept | 22 Sep | none | none | 9925 | 34554 |
| | 2 | 13 May | 24 May | 11 Jun | 16 Aug | 30 Aug | 19 Sep | none | none | 6005 | 17235 |
| Coal tit (Periparus ater; PERATE) | 1 | 13 May | 24 May | 11 Jun | 17 Sep | 25 Sept | 13 Oct | rm3 | none | - | 167115 (24603) |

| Species | Zone | Spr 5% | Spr 50% | Spr 95% | Aut 5% | Aut 50% | Aut 95% | Proc Spr | Proc Aut | n NCL Spr | n NCL Aut (n Hanko) |
|---|---|---|---|---|---|---|---|---|---|---|---|
| Great cormorant (Phalacrocorax carbo; PHACAR) | 1 | 26 Mar | 20 Apr | 27 May | 18 Jul | 27 Aug | 11 Oct | rm3 | rm3 | 629722 | 1866766 |
| | 2 | 26 Mar | 19 Apr | 26 May | 12 Jul | 08 Aug | 27 Sep | rm3 | rm3 | 569071 | 942334 |
| | 3 | 03 Apr | 22 Apr | 24 May | 26 Jul | 17 Aug | 05 Oct | rm3 | rm3 | 25793 | 12955 |
| | 4 | 07 Apr | 28 Apr | 25 May | 24 Jul | 06 Sept | 07 Oct | rm3 | rm3 | 4675 | 5709 |
| Common redstart (Phoenicurus phoenicurus; PHOPHO) | 1 | 28 Apr | 11 May | 26 May | 20 Aug | 07 Sept | 03 Oct | none | rm3 | 24192 | 8090 (1180) |
| | 2 | 27 Apr | 12 May | 30 May | 16 Aug | 06 Sept | 28 Sep | A1-3(H),rm3 | A3(B),rm3 | 11511 | 3736 |
| | 3 | 30 Apr | 14 May | 02 Jun | 12 Aug | 30 Aug | 21 Sep | A1-2(H),rm3 | rm3 | 1723 | 546 |
| | 4 | 03 May | 19 May | 06 Jun | 08 Aug | 26 Aug | 17 Sep | A1(H),rm3 | rm5 | 1825 | 669 |
| Chiffchaff (Phylloscopus collybita; PHYCOL) | 1 | 14 Apr | 03 May | 29 May | 09 Sep | 28 Sept | 17 Oct | rm3 | none | 21854 | 41139 (1451) |
| | 2 | 17 Apr | 06 May | 01 Jun | 02 Sep | 21 Sept | 12 Oct | rm3 | none | 21975 | 33349 |
| | 3 | 21 Apr | 14 May | 08 Jun | 03 Sep | 20 Sept | 09 Oct | rm5 | none | 2093 | 2345 |
| | 4 | 22 Apr | 14 May | 04 Jun | 22 Aug | 19 Sept | 11 Oct | A1(H),rm3 | rm5 | 939 | 519 |
| Wood warbler (Phylloscopus sibilatrix; PHYSIB) | 1 | 01 May | 16 May | 01 Jun | 27 Jul | 12 Aug | 06 Sep | A1(H,B) | rm3 | 16274 | 1971 (891) |
| | 2 | 05 May | 20 May | 08 Jun | 15 Jul | 31 Jul | 23 Aug | A1(H,B) | rm3 | 15332 | 1001 |
| | 3 | 12 May | 26 May | 10 Jun | 12 Jul | 24 Jul | 19 Aug | A1(H,B) | rm3 | 1267 | 79 |
| Willow warbler (Phylloscopus trochilus; PHYLUS) | 1 | 01 May | 18 May | 04 Jun | 05 Aug | 29 Aug | 21 Sep | rm3 | rm3 | 79304 | 63766 (17783) |
| | 2 | 30 Apr | 16 May | 08 Jun | 27 Jul | 27 Aug | 23 Sep | A1(H),rm3 | rm3 | 38237 | 41682 |
| | 3 | 05 May | 21 May | 10 Jun | 01 Aug | 27 Aug | 22 Sep | rm3 | rm3 | 6061 | 5718 |
| | 4 | 06 May | 24 May | 15 Jun | 27 Jul | 19 Aug | 19 Sep | A1(H),rm3 | rm5 | 3255 | 6821 |
| Golden plover (Pluvialis apricaria; PLUAPR) | 1 | 20 Apr | 04 May | 14 May | 31 Jul | 01 Sept | 06 Oct | none | rm5 | 457299 | 83808 |
| | 2 | 26 Apr | 07 May | 18 May | 28 Jul | 08 Sept | 04 Oct | none | rm3 | 805299 | 89364 |
| | 3 | 29 Apr | 13 May | 21 May | 03 Aug | 19 Sept | 06 Oct | none | rm3 | 43781 | 7269 |
| | 4 | 06 May | 13 May | 28 May | 21 Jul | 25 Aug | 02 Oct | A1(H) | rm3 | 21995 | 2491 |
| Kentish plover (Pluvialis squatarola; PLUSQU) | 1 | 22 May | 28 May | 02 Jun | 28 Jul | 15 Aug | 04 Oct | none | A2(B),rm3 | 329872 | 17486 |
| Great crested grebe (Podiceps cristatus; PODCRI) | 1 | 09 Apr | 27 Apr | 23 May | 18 Jul | 13 Sept | 24 Oct | rm3 | rm3 | 147070 | 145397 |
| | 2 | 13 Apr | 29 Apr | 22 May | 11 Jul | 02 Sept | 21 Oct | rm3 | rm3 | 165852 | 296824 |
| Dunnock (Prunella modularis; PRUMOD) | 1 | 01 Apr | 17 Apr | 08 May | 02 Sep | 20 Sept | 13 Oct | none | none | 18628 | 98028 (1718) |
| | 2 | 08 Apr | 22 Apr | 11 May | 25 Aug | 14 Sept | 05 Oct | none | none | 18008 | 56868 |
| | 3 | 15 Apr | 27 Apr | 18 May | 26 Aug | 15 Sept | 06 Oct | none | rm3 | 2114 | 4143 |
| | 4 | 21 Apr | 03 May | 28 May | 27 Aug | 18 Sept | 07 Oct | rm3 | rm3 | 1640 | 4353 |
| Goldcrest (Regulus regulus; REGREG) | 1 | 25 Mar | 12 Apr | 03 May | 09 Sep | 04 Oct | 23 Oct | rm3 | rm3 | 21018 | 240769 (28953) |
| | 2 | 29 Mar | 15 Apr | 12 May | 07 Sep | 29 Sept | 20 Oct | rm3 | rm3 | 9507 | 214545 |
| | 3 | 29 Mar | 18 Apr | 13 May | 04 Sep | 30 Sept | 17 Oct | rm3 | rm3 | 876 | 4607 |
| | 4 | 30 Mar | 17 Apr | 16 May | 26 Aug | 24 Sept | 13 Oct | A1(H),rm3 | rm3 | 607 | 2288 |
| Sand martin (Riparia riparia; RIPRIP) | 1 | 04 May | 23 May | 13 Jun | 16 Jul | 11 Aug | 08 Sep | rm3 | rm5 | 17153 | 11906 |
| | 2 | 07 May | 26 May | 13 Jun | 12 Jul | 29 Jul | 29 Aug | rm3 | rm5 | 53902 | 19874 |
| | 3 | 12 May | 29 May | 15 Jun | 09 Jul | 29 Jul | 25 Aug | rm3 | rm5 | 20567 | 17581 |
| | 4 | 15 May | 30 May | 18 Jun | 10 Jul | 27 Jul | 21 Aug | rm3 | rm5 | 28030 | 11619 |
| Whinchat (Saxicola rubetra; SAXTRA) | 1 | 28 Apr | 13 May | 31 May | 07 Aug | 24 Aug | 14 Sep | rm3 | rm3 | 16791 | 25028 (205) |
| | 2 | 02 May | 16 May | 03 Jun | 01 Aug | 20 Aug | 11 Sep | rm3 | rm3 | 14706 | 22307 |
| | 3 | 04 May | 20 May | 08 Jun | 02 Aug | 18 Aug | 08 Sep | rm3 | rm3 | 1672 | 1901 |
| | 4 | 07 May | 21 May | 09 Jun | 01 Aug | 16 Aug | 05 Sep | rm3 | rm3 | 1121 | 996 |

| Species | Zone | Spr 5% | Spr 50% | Spr 95% | Aut 5% | Aut 50% | Aut 95% | Proc Spr | Proc Aut | n NCL Spr | n NCL Aut (n Hanko) |
|---|---|---|---|---|---|---|---|---|---|---|---|
| Eurasian woodcock (Scolopax rusticola; SCORUS) | 1 | 23 Mar | 09 Apr | 28 Apr | 25 Sep | 21 Oct | 13 Nov | rm3 | rm3 | 8155 | 2784 |
| | 2 | 02 Apr | 19 Apr | 09 May | 21 Sep | 12 Oct | 04 Nov | rm3 | rm3 | 8472 | 3273 |
| | 3 | 11 Apr | 29 Apr | 19 May | 14 Sep | 07 Oct | 28 Oct | rm3 | rm3 | 1230 | 972 |
| | 4 | 17 Apr | 07 May | 25 May | 13 Sep | 04 Oct | 25 Oct | rm3 | rm3 | 921 | 653 |
| Eider (Somateria mollissima; SOMMOL) | 1 | 26 Mar | 14 Apr | 05 May | 30 Jun | 23 Aug | 11 Oct | rm3 | rm3 | 5059980 | 2593555 |
| | 2 | 27 Mar | 13 Apr | 03 May | 02 Jul | 16 Aug | 08 Oct | rm3 | rm3 | 1312210 | 549704 |
| Common tern (Sterna hirundo; STEHIR) | 1 | 17 Apr | 05 May | 23 May | 12 Jul | 31 Jul | 26 Aug | rm3 | rm3 | 68769 | 74088 |
| | 2 | 26 Apr | 14 May | 03 Jun | 10 Jul | 01 Aug | 24 Aug | rm3 | rm3 | 99850 | 155314 |
| | 3 | 02 May | 16 May | 02 Jun | 03 Jul | 29 Jul | 03 Sep | rm3 | rm3 | 4476 | 5232 |
| Arctic tern (Sterna paradisaea; STEAEA) | 1 | 30 Apr | 19 May | 04 Jun | 04 Jul | 17 Jul | 07 Aug | rm3 | rm3 | 137548 | 63693 |
| | 2 | 29 Apr | 19 May | 04 Jun | 01 Jul | 17 Jul | 07 Aug | rm3 | rm3 | 76230 | 45473 |
| | 3 | 10 May | 24 May | 09 Jun | 09 Jul | 20 Jul | 16 Aug | rm3 | rm3 | 4561 | 2022 |
| | 4 | 12 May | 26 May | 10 Jun | 14 Jul | 27 Jul | 13 Aug | rm3 | rm3 | 4844 | 1745 |
| Starling (Sturnus vulgaris; STUVUL) | 1 | 19 Mar | 03 Apr | 19 Apr | 01 Jul | 28 Aug | 12 Oct | none | rm3 | 397973 | 4055430 (161) |
| | 2 | 24 Mar | 08 Apr | 22 Apr | 02 Jul | 31 Aug | 09 Oct | none | rm3 | 233159 | 2252093 |
| | 3 | 24 Mar | 16 Apr | 30 Apr | 01 Jul | 24 Aug | 02 Oct | none | rm5 | 11230 | 183420 |
| | 4 | 01 Apr | 20 Apr | 08 May | 23 Jun | 10 Aug | 02 Oct | rm3 | rm5 | 3485 | 54796 |
| Blackcap (Sylvia atricapilla; SYLATR) | 1 | 28 Apr | 17 May | 10 Jun | 22 Aug | 18 Sept | 18 Oct | rm3 | rm3 | 25828 | 8785 (1758) |
| | 2 | 05 May | 26 May | 18 Jun | 23 Aug | 12 Sept | 25 Oct | rm3 | rm3 | 17157 | 6659 |
| Garden warbler (Sylvia borin; SYLBOR) | 1 | 13 May | 27 May | 12 Jun | 03 Aug | 29 Aug | 23 Sep | rm3 | rm3 | 16623 | 4217 (4857) |
| | 2 | 14 May | 29 May | 17 Jun | 01 Aug | 25 Aug | 18 Sep | rm3 | rm3 | 15385 | 3911 |
| | 3 | 17 May | 31 May | 18 Jun | 02 Aug | 30 Aug | 18 Sep | rm3 | rm5 | 1407 | 308 |
| | 4 | 25 May | 11 Jun | 28 Jun | 02 Aug | 24 Aug | 17 Sep | rm3 | rm3 | 1247 | 200 |
| Common whitethroat (Sylvia communis; SYLCOM) | 1 | 07 May | 23 May | 09 Jun | 31 Jul | 17 Aug | 09 Sep | rm3 | rm3 | 24007 | 9507 (1002) |
| | 2 | 09 May | 25 May | 13 Jun | 31 Jul | 19 Aug | 09 Sep | rm3 | rm3 | 12539 | 4133 |
| | 3 | 15 May | 30 May | 16 Jun | 01 Aug | 16 Aug | 08 Sep | A1(H,B),rm3 | rm3 | 848 | 84 |
| Lesser whitethroat (Sylvia curruca; SYLCUR) | 1 | 03 May | 17 May | 02 Jun | 03 Aug | 27 Aug | 20 Sep | none | rm3 | 39940 | 15989 (3308) |
| | 2 | 04 May | 17 May | 05 Jun | 27 Jul | 25 Aug | 18 Sep | none | rm3 | 15084 | 10310 |
| | 3 | 07 May | 21 May | 11 Jun | 04 Aug | 26 Aug | 16 Sep | rm3 | rm3 | 1462 | 738 |
| Spotted redshank (Tringa erythropus; TRIERY) | 1 | 01 May | 08 May | 16 May | 13 Jul | 06 Aug | 04 Sep | none | rm3 | 27092 | 16331 |
| | 2 | 03 May | 10 May | 16 May | 11 Jul | 04 Aug | 02 Sep | none | rm3 | 53054 | 18989 |
| | 3 | 03 May | 11 May | 16 May | 12 Jul | 04 Aug | 05 Sep | none | rm3 | 19422 | 5936 |
| | 4 | 07 May | 13 May | 21 May | 16 Jul | 06 Aug | 05 Sep | none | rm3 | 5798 | 1457 |
| Wood sandpiper (Tringa glareola; TRIGLA) | 1 | 02 May | 09 May | 17 May | 23 Jun | 24 Jul | 18 Aug | none | rm3 | 370766 | 309380 |
| | 2 | 03 May | 10 May | 19 May | 25 Jun | 22 Jul | 14 Aug | none | rm3 | 461239 | 250047 |
| | 3 | 06 May | 13 May | 21 May | 23 Jun | 24 Jul | 14 Aug | none | rm3 | 66450 | 46184 |
| | 4 | 07 May | 14 May | 28 May | 24 Jun | 23 Jul | 18 Aug | none | rm3 | 25679 | 10479 |
| Common greenshank (Tringa nebularia; TRINEB) | 1 | 23 Apr | 02 May | 11 May | 27 Jun | 01 Aug | 04 Sep | none | rm3 | 75007 | 63599 |
| | 2 | 26 Apr | 03 May | 13 May | 23 Jun | 25 Jul | 28 Aug | none | rm3 | 135885 | 82759 |
| | 3 | 28 Apr | 08 May | 16 May | 21 Jun | 26 Jul | 28 Aug | none | rm3 | 17065 | 11178 |
| | 4 | 02 May | 10 May | 22 May | 11 Jun | 25 Jul | 28 Aug | none | rm3 | 6978 | 3678 |
| Green sandpiper (Tringa ochropus; TRIOCH) | 1 | 08 Apr | 20 Apr | 03 May | 05 Jun | 18 Jul | 16 Aug | none | rm3 | 33848 | 15611 |
| | 2 | 13 Apr | 24 Apr | 08 May | 01 Jun | 07 Jul | 08 Aug | none | rm3 | 55131 | 11135 |
| | 3 | 21 Apr | 30 Apr | 12 May | 01 Jun | 28 Jun | 06 Aug | none | rm3 | 4469 | 1022 |
| | 4 | 23 Apr | 05 May | 18 May | 01 Jun | 19 Jun | 05 Aug | none | rm3 | 2228 | 1006 |

| Species | Zone | Spr 5% | Spr 50% | Spr 95% | Aut 5% | Aut 50% | Aut 95% | Proc Spr | Proc Aut | n NCL Spr | n NCL Aut (n Hanko) |
|---|---|---|---|---|---|---|---|---|---|---|---|
| Common redshank (Tringa totanus; TRITOT) | 1 | 12 Apr | 30 Apr | 16 May | 30 Jun | 25 Jul | 19 Aug | rm3 | A3(B) | 29878 | 19869 |
| Eurasian wren (Troglodytes troglodytes; TROTRO) | 1 | 28 Mar | 23 Apr | 14 May | 17 Sep | 09 Oct | 04 Nov | rm3 | rm3 | 10229 | 35879 (1709) |
| | 2 | 04 Apr | 30 Apr | 20 May | 31 Aug | 01 Oct | 31 Oct | rm3 | rm3 | 7874 | 9841 |
| Redwing (Turdus iliacus; TURILI) | 1 | 01 Apr | 12 Apr | 25 Apr | 25 Sep | 13 Oct | 28 Oct | rm3 | rm3 | 204585 | 95233 (411) |
| | 2 | 08 Apr | 23 Apr | 05 May | 15 Sep | 02 Oct | 17 Oct | rm3 | rm3 | 173117 | 357454 |
| | 3 | 19 Apr | 28 Apr | 09 May | 10 Sep | 01 Oct | 17 Oct | none | rm3 | 18367 | 29162 |
| | 4 | 19 Apr | 02 May | 16 May | 08 Sep | 26 Sept | 14 Oct | rm3 | rm3 | 12836 | 16561 |
| Common blackbird (Turdus merula; TURMER) | 1 | 15 Mar | 31 Mar | 20 Apr | 26 Sep | 19 Oct | 16 Nov | rm3 | rm3 | 73054 | 83955 (602) |
| | 2 | 16 Mar | 04 Apr | 20 Apr | 20 Sep | 12 Oct | 13 Nov | rm3 | rm3 | 73890 | 46243 |
| | 3 | 20 Mar | 10 Apr | 27 Apr | 19 Sep | 10 Oct | 02 Nov | rm3 | rm3 | 5633 | 2701 |
| Song thrush (Turdus philomelos; TURPHI) | 1 | 03 Apr | 22 Apr | 10 May | 10 Sep | 30 Sept | 19 Oct | rm3 | rm3 | 63138 | 62907 (1259) |
| | 2 | 07 Apr | 24 Apr | 11 May | 06 Sep | 27 Sept | 17 Oct | rm3 | rm3 | 25984 | 38552 |
| | 3 | 19 Apr | 02 May | 16 May | 01 Sep | 26 Sept | 16 Oct | rm3 | rm3 | 3222 | 2624 |
| | 4 | 22 Apr | 05 May | 22 May | 31 Aug | 21 Sept | 14 Oct | rm3 | rm3 | 1623 | 1686 |
| Fieldfare (Turdus pilaris; TURPIL) | 1 | 02 Apr | 14 Apr | 26 Apr | 25 Sep | 19 Oct | 12 Nov | none | rm3 | 638010 | 2536440 (555) |
| | 2 | 07 Apr | 20 Apr | 01 May | 14 Sep | 08 Oct | 31 Oct | none | rm3 | 892466 | 4755007 |
| | 3 | 17 Apr | 27 Apr | 08 May | 17 Sep | 05 Oct | 01 Nov | none | rm3 | 51964 | 482308 |
| | 4 | 18 Apr | 30 Apr | 15 May | 10 Sep | 30 Sept | 21 Oct | none | rm3 | 28394 | 130175 |
| Mistle thrush (Turdus viscivorus; TURVIS) | 1 | 25 Mar | 11 Apr | 03 May | 23 Sep | 06 Oct | 22 Oct | none | rm3 | 31264 | 81213 (112) |
| | 2 | 01 Apr | 19 Apr | 08 May | 10 Sep | 02 Oct | 22 Oct | none | rm3 | 46462 | 29033 |
| | 3 | 12 Apr | 27 Apr | 12 May | 08 Sep | 02 Oct | 21 Oct | rm3 | rm3 | 6099 | 2513 |
| | 4 | 18 Apr | 03 May | 20 May | 05 Sep | 26 Sept | 18 Oct | rm3 | rm3 | 4884 | 4167 |
| Lapwing (Vanellus vanellus; VANVAN) | 1 | 13 Mar | 04 Apr | 20 Apr | 06 Jul | 26 Aug | 12 Oct | rm3 | rm3 | 856681 | 1432137 |
| | 2 | 18 Mar | 07 Apr | 24 Apr | 03 Jul | 29 Jul | 17 Sep | rm3 | rm3 | 1305498 | 456784 |
| | 3 | 28 Mar | 15 Apr | 02 May | 02 Jul | 31 Jul | 05 Sep | rm3 | rm3 | 73630 | 9749 |
| | 4 | 06 Apr | 22 Apr | 09 May | 04 Jul | 27 Jul | 26 Aug | rm3 | rm3 | 19868 | 2578 |
